# Research on the Elastocaloric Effects Performance of the Fe14.6Ni(at%) Alloy via Successive and Pulsing Loading Methods: A Molecular Dynamics Study

**DOI:** 10.3390/ma19183909

**Published:** 2026-09-15

**Authors:** Xianfa Li, Yulin Gong, Yabin Yang, Guoqiang Chen, Long Zhou, Jianli Kang, Yanpeng Wang, Chong Lv

**Affiliations:** 1 School of Mechanical and Power Engineering, Henan Polytechnic University, Jiaozuo 454099, China; 2 Henan Tianhai Electric Co., Ltd., Hebi 410611, China; 3 School of Computer Science and Artificial Intelligence, Henan Finance University, Zhengzhou 450014, China; 4 School of Emergency Management, Henan Polytechnic University, Jiaozuo 454099, China

**Keywords:** Fe14.6Ni(at%) alloy, eCEs performance, molecular dynamics, loading methods, cyclic stability

## Abstract

Successive and pulsing loading methods were employed to evaluate the elastocaloric effects (eCEs) performance of the Fe14.6Ni(at%) alloy at 293 K by molecular dynamics (MD) simulation. The study included investigation of the loading methods and the cyclic stability of eCEs performance. The study of the loading methods indicated that the pulse cyclic loading method, compared with the successive loop loading method, was the optimal option due to the steady evolution processes of the parameters. Finally, the research on cyclic stability demonstrated that the Fe14.6Ni(at%) alloy remained sensitive to the external stimulus even after 80,414 cycles. This work proposes a promising strategy for optimizing the eCEs performance of the Fe14.6Ni(at%) alloy and demonstrates its great industrial potential.

## 1. Introduction

Recently, elastocaloric refrigeration alloys have attracted many scholars’ attention as exigent solid-state refrigeration materials to replace traditional vapor compression gas refrigerant, due to their advantages of high thermal efficiency, reusability, and environmental friendliness [1,2,3].

Ossmer et al. [4,5] investigated the time-resolved strain of magnetron-sputtered superelastic TiNi films of 20 um thickness during tensile stress cycling for different strain rates. They discussed the prospects of SMA film-based elastocaloric cooling. The results showed that the investigated film samples exhibited an undercooling of −16 K upon mechanical unloading under adiabatic conditions at a strain rate of 0.2 s^−1^ and the coefficient of performance (COP) was determined to be 7.7. Tušek et al. [6] trained the superelastic Ni-Ti wire with 400 loading/unloading cycles at different temperatures to study the impact of the training temperature on the adiabatic temperature change. The results showed that the largest measured ∆T_adi_ during loading was 25 K, corresponding to a 21 K change during unloading (at 322 K). Hou et al. [7] used a localized molten environment and near-eutectic mixing of elemental powders to prepare the thermodynamically efficient, low-hysteresis elastocaloric Ni-Ti alloy by using additive manufacturing. The results demonstrated that the microstructure of the Ni-Ti alloy allowed extremely small hysteresis in quasi-linear stress–strain behaviors, enhancing the material’s efficiency by a factor of four to seven and obtaining a repeatable elastocaloric performance over 1 million cycles. Czernuszewicz et al. [8] described the concept of a novel composite, wherein an active NiTi layer was embedded into a polymer support structure, such that the elastocaloric material was entirely in compression when the assembly was subjected to bending. The results showed that the active layer achieved a 8.1 K temperature change at 2.5% compressive strain without buckling and the composite maintained mechanical integrity without degradation of the elastocaloric effect after 10,000 cycles at 2% compressive strain. Guo et al. [9] investigated the superelastic behavior and elastocaloric effect of a [001]-textured Ni50Mn23Ga25Cu2 polycrystal below room temperature (256–294 K). The results showed that adiabatic temperature change caused by rapid unloading of uniaxial stress reached −4.3 K at 259 K and a small stress hysteresis of 14 MPa was observed during the experimental process. In addition, because of the low hysteresis energy loss (0.37 MJ m^−3^) stemming from the textured structure, the COP of the alloy was calculated to be 18, which indicated that it was a promising elastocaloric candidate for low-temperature refrigeration. Xuan et al. [10] comprehensively investigated the elastocaloric properties of Ni_40_Fe*_x_*Co_10-*x*_Mn_32_Al_18_ (x = 2, 4, 6 at%) polycrystalline alloys. It was found that a large temperature change of 6.1 K was obtained by applying uniaxial stress of 400 MPa and a temperature change of about −5.2 K was maintained as almost constant after 100 stress cycles in the Ni_40_Fe_4_Co_6_Mn_32_Al_18_ alloy. Cao et al. [11] prepared a Mn_49_Ni_41_Sn_9.4_Gd_0.6_ alloy by replacing Sn with Gd and systematically investigated the elastocaloric effect (eCE) of the Mn_49_Ni_41_Sn_9.4_Gd_0.6_ ferromagnetic shape memory alloy. It was found that a large ∆T_adi_ of 11.4 K was obtained by the stress (500 MPa) removal and a constant temperature change of 9.6 K showed no apparent degradation under the compressive stress of 400 MPa during 200 cycles for the Mn_49_Ni_41_Sn_9.4_Gd_0.6_ alloy. Bonnot et al. [12] studied the elastocaloric effect in the vicinity of the martensitic transition of the Cu-Zn-Al alloy by experimental methods and discussed the possibility of using these materials for mechanical refrigeration. The results showed that the entropy change associated with the whole transformation (∆St) coincided with the experiments. Imran et al. [13,14] fabricated a Ni_53.5_Fe_19.9_Ga_26.6_ foam with 53% porosity by a replication casting technique and investigated its elastocaloric properties. The results illustrated that the Ni-Fe-Ga foam expressed reversible superelasticity with a strain 3.9% above the austenite finishing temperature and also exhibited a ∆T_adi_ of 2.8 K. The compressive cyclic test revealed that the foam showed better cyclic performance up to 100 cycles, over bulk samples under the same loading effect, in which the enhanced cyclic stability in the foam was achieved due to pores and narrow hysteresis energy loss. Panchenko et al. [15] systematically investigated the effects of stress-induced martensite aging (SIM-aging) in twinned L1(0)-martensite on the functional properties of [001](A)-oriented Ni_51_Fe_18_Ga_27_Co_4_ (at%) single crystals. It was found that SIM-aging of as-grown single crystals with gamma-phase precipitates at T-SIM = 448 K for 1 h, under a compressive stress sigma (SIM) of 700 MPa along the [001](A)parallel to the [110](M)-direction induced a two-way shape memory effect with a reversible strain of up to −3.4(±0.3)% and that the SIM-aged crystals demonstrated a 1.5 to 2 times decrease in thermal and stress hysteresis compared to the as-grown crystals. The experimental method has been widely adopted and has achieved fruitful results in the study of the eCEs of Ni-Ti-based [6,15,16,17], Ni-Mn-based [2,9], Cu-based [18], and Fe-based alloys [13,14].

However, investigating the eCEs by experimental methods also faces great challenges [4,6,19,20,21]. Firstly, the adiabatic environment is achieved by rapidly loading and unloading, which requires a quick response from the tensile testing machine [4,19]. Secondly, the ∆T_adi_ is usually measured by a thermocouple [6] or an infrared camera [20]. The thermocouple, as touching sensors, would change the thermos boundary due to heat conduction [21]. Though an infrared camera could measure the temperature without contact with the specimens, the subtle changes in the ∆T_adi_ would place demand on the temperature resolution of the device [8]. Thirdly, a transmission electron microscope (TEM) may be adopted and retrofitted to study the evolution of the crystal structure by experimental methods, but this has a high expenditure and requires advanced technology [18]. These restrictions greatly limited the development and understanding of the eCEs performance, such as the ∆T_adi_, cyclic life, stress hysteresis, and COP, of elastocaloric refrigeration alloys [9,14].

Luckily, the prosperous development of MD makes it possible to study the eCEs performance at the atomic scale [22,23,24]. Studying eCEs performance through MD simulation can easily create adiabatic environmental conditions [25]. It provides a dynamic evaluation of the parameters’ evolution, such as stress, temperature, total energy, and crystal structure [26]. It breaks through the limits of the experimental methods and has become a new research method. More importantly, the crystal structure of the elastocaloric refrigeration alloy can be viewed and pictured directly [27]. Due to these advantages, MD simulation methods have been conducted widely and fruitful achievements have been reported. Zhang et al. [28] performed MD simulation to study the underlying atomistic mechanism of the pseudoelastic behavior of zirconia nanopillars. It was found that irreversible deformation appeared because of crack propagation, owing to the shear deformation alongside the phase interfaces. The energy and stress analysis demonstrated that the mechanism responsible for the pseudoelasticity was the high surface-stress-induced internal stresses at the nanometer scale, which promoted the understanding of the pseudoelasticity of zirconia nanopillar on an atomic scale. Wang et al. [29] used molecular dynamics simulation to study the mechanical behavior and phase transformation of a nanocrystalline NiTi shape memory alloy with a gradient structure. In the gradient structure, surface layers possessed coarse grain sizes of 6, 9, 12, and 15 nm, respectively, and the middle layer had an average grain size of 20 nm. It was found that the amorphous phase and grain boundaries played a vital impact on the superelasticity of the NiTi alloy and the degradation of the superelasticity mainly resulted from the grain boundaries and plastic deformation of the amorphous phase. Wu et al. [30] conducted molecular dynamics simulations to investigate the deposition behavior of composite graphene nanoplatelets (GNPs)-Ni particles at various velocities and with different graphene contents. The results indicated that GNPs impede the plastic deformation of the metallic particle and also impede the stress transfer to it, simultaneously limiting metallurgical bonding at the interface with the substrate, which deepened the understanding of how graphene nanoplate–metal composites form. Zhang et al. [27] observed an unconventional stress–strain relationship of two-stage linear elastic deformation of [001]-oriented tetragonal zirconia nanopillars during the tensile loading process of a molecular dynamics simulation. The results showed that this phenomenon was attributed to a phase transformation from a tetragonal structure to a monoclinic one, which deepened the understanding of the tetragonal-to-monoclinic transformation and the mechanical behavior of zirconia at the nanoscale. Li et al. [31,32] simulated the mechanical behaviors of the Fe-Ni and Fe-Co elastocaloric refrigeration alloys by the MD method. The evaluation of the mechanical behaviors of the alloy showed that a novel stress–strain relationship of two-stage linear elastic deformation occurred during the uniaxial tensile processes and the mechanical properties of the Fe14.6Ni (at%) and Fe9.5Co (at%) alloys were dependent on the strain rate and environmental temperature. Li et al. [33] investigated the mechanical behaviors of the Fe14.6Ni (at%) nanocrystalline elastocaloric refrigeration alloy in an adiabatic system through MD simulation. The results showed that the mechanical behaviors were unsteady compared to the open thermodynamic system. Furthermore, the evaluation of the total kinetic energy and the crystal structure illustrated that the origin of the unsteady mechanical behavior of the Fe14.6Ni(at%) alloy in the adiabatic system was the macro-performance of the interaction between the crystal structure and the total kinetic energy evolution.

Until now, as far as we know, little research has been reported about the eCEs performance by MD simulation methods [27,28,29,31,32,33]. The Fe14.6Ni(at%) alloy, as a classic martensite transformation alloy, has a huge latent heat effect [32], of which the martensite transformation enthalpy is as large as 34.43 J·g^−1^ [33]. Successive and pulsing loading methods were employed to investigate the eCEs performance of the Fe14.6(at%)Ni alloy at 293 K by MD simulation in the investigation. The successive and pulsing loading methods were researched in order based on the study of the mechanical behaviors and the temperature evolution. Finally, symmetrical pulse cyclic loads were imposed to investigate the cyclic stability of the eCEs performance of the Fe14.6(at%)Ni alloy.

## 2. Simulation Settings

The MD simulations were performed using the package Large-Scale Atomic/Molecular Massively Parallel Simulator (LAMMPS 2020) [34] and the Open Visualization Tool (OVITO 2020) software was adopted to view the process [35]. The EAM potential function developed by Bonny et al. [36] was selected as the force field to simulate the uniaxial tensile process of the Fe14.6Ni (at %) alloy.

As for the system settings, the periodic boundary conditions were chosen in the three directions of Cartesian coordinates to eliminate the dimensional boundary effects. The integrated Newton’s equations of motion used the Velocity-Verlet algorithm [37]. The Canonical NVT ensemble was employed in the simulation to simulate the adiabatic system. Start and stop temperature were both set as 293 K. The temperature damping parameter was set at 0.2 ps. The damping parameter was 0.2, which is suitable for Fe-based elastocaloric alloys. The system temperature was controlled by the Nose–Hoover thermostat [38,39]. The relaxation coefficient was set as 0.2 to prevent the temperature rising and falling rapidly. Before the uniaxial tensile simulation began, an energy minimization with a conjugate gradient (CG) algorithm was conducted to balance the system. The method for calculating the system’s temperature was shown as follows:

(1) $$T=\frac{2}{3Nk_{b}}\sum_{i}\frac{p_{i}^{2}}{m_{i}}$$

in which T is temperature in Kelvin, *N* is the number of all atoms in the system, *k_b_* is the Boltzmann constant, *p_i_* is the momentum of the *i*th atom and the *m_i_* is the mass of the *i*th atom.

For the MD simulation model, the Fe14.6Ni(at%) solid solution alloy, of which the space group was the body-centered cubic (BCC) structure of Im-3m 229 with a lattice constant of 2.863 Å, was selected to perform the MD simulation. The shape of the model was a rectangle containing about 4000 atoms. The length, width, and height were 56.94 Å, 28.47 Å, and 28.47 Å, respectively. The Fe14.6Ni(at%) alloy model was placed in a vacuum box and the mass center was located at the origin of the coordinates. The box size was the same as the volume of the model so that the system could easily and quickly reach equilibrium. The correctness of the model and the applicability of the EAM potential function have been verified in the previous study by experimental methods [32,33].

In order to evaluate the stability of the system energy of the Fe14.6Ni(at%) alloy during the simulations, the total energy was calculated [31,32]. Furthermore, common neighborhood analysis (CNA) was utilized to describe the evolutions of the phase contents and the crystal structure during the MD simulations [40,41,42,43].

## 3. Results and Discussion

Referring to previous investigations [33], the conclusion was drawn that the application of a preload was required. The strain interval of the preload process was determined to be [0, 0.108] and the duration of the preload process was 0.360 ps. Based on the results the loading methods, including the successive loop loading method and the pulse cyclic loading method, were explored to evaluate the eCEs performance of the Fe14.6Ni(at%) alloy at 293 K.

### 3.1. The Study of the eCEs Performance Adopting a Successive Cyclic Loading Method

**Figure 1:**
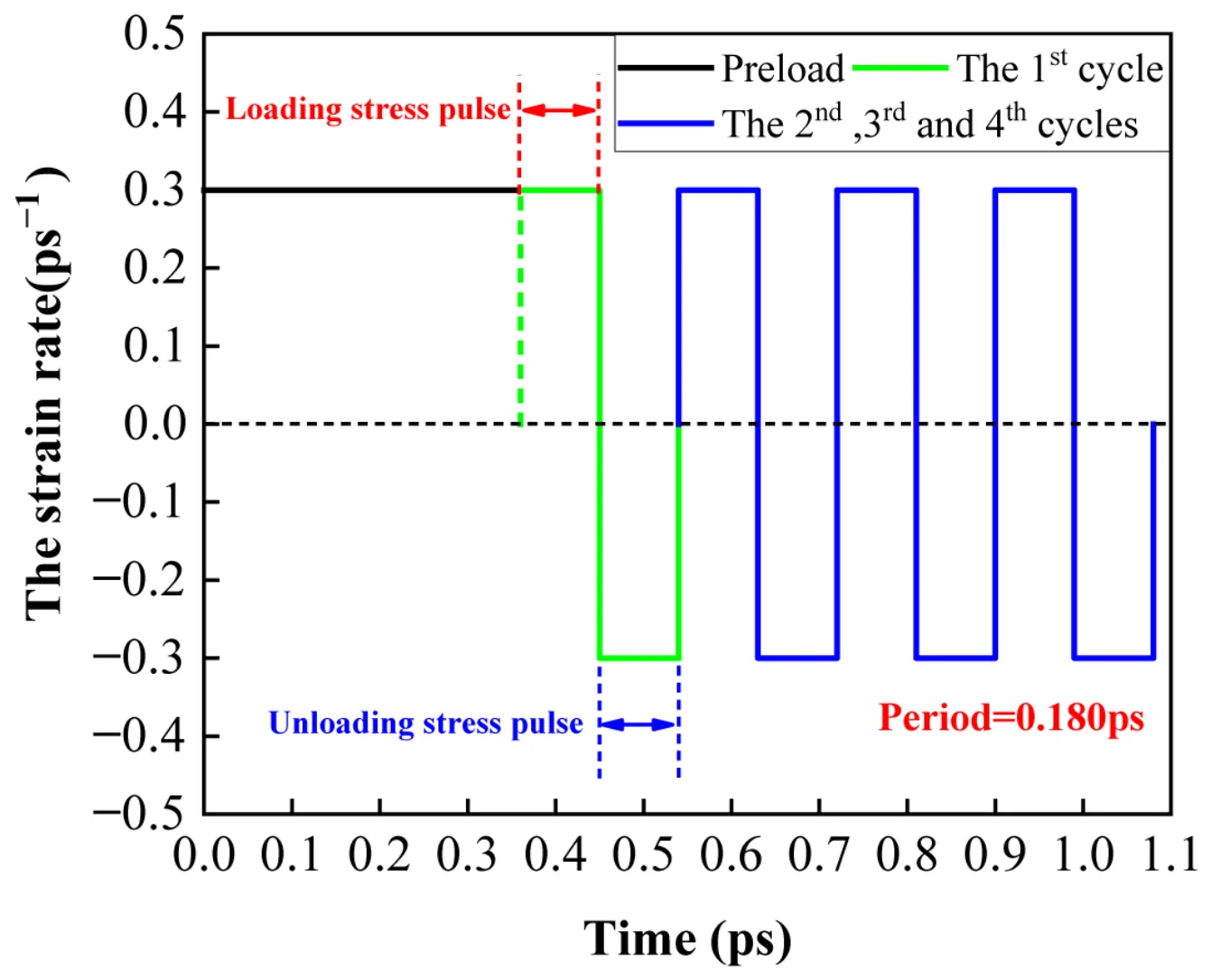
The stress pulses during the preload and the four successive cyclic loading processes.

This section investigates the eCEs performance of the Fe14.6Ni(at%) alloy under the successive cyclic loading condition, shown in Figure S1 and Table S1 in the Supplementary Materials. Based on the above results, a preload and four-time successive cyclic loading were imposed on the Fe14.6Ni(at%) alloy. From a published study [33], as listed in Table S1 in the Supplementary Materials, it was determined that the strain interval of the phase transformation stage was [0.108, 0.177]. Hence, the subinterval, from 0.108 to 0.135, was selected as the phase transformation interval to research successive cyclic loading processes at 293 K. The strain rates during the loading and unloading processes were both controlled at 0.3 ps^−1^. The loading and unloading processes both lasted for 0.090 ps. Figure 1 depicts the stress pulses during the preload and the four successive cyclic loading processes.

**Figure 2:**
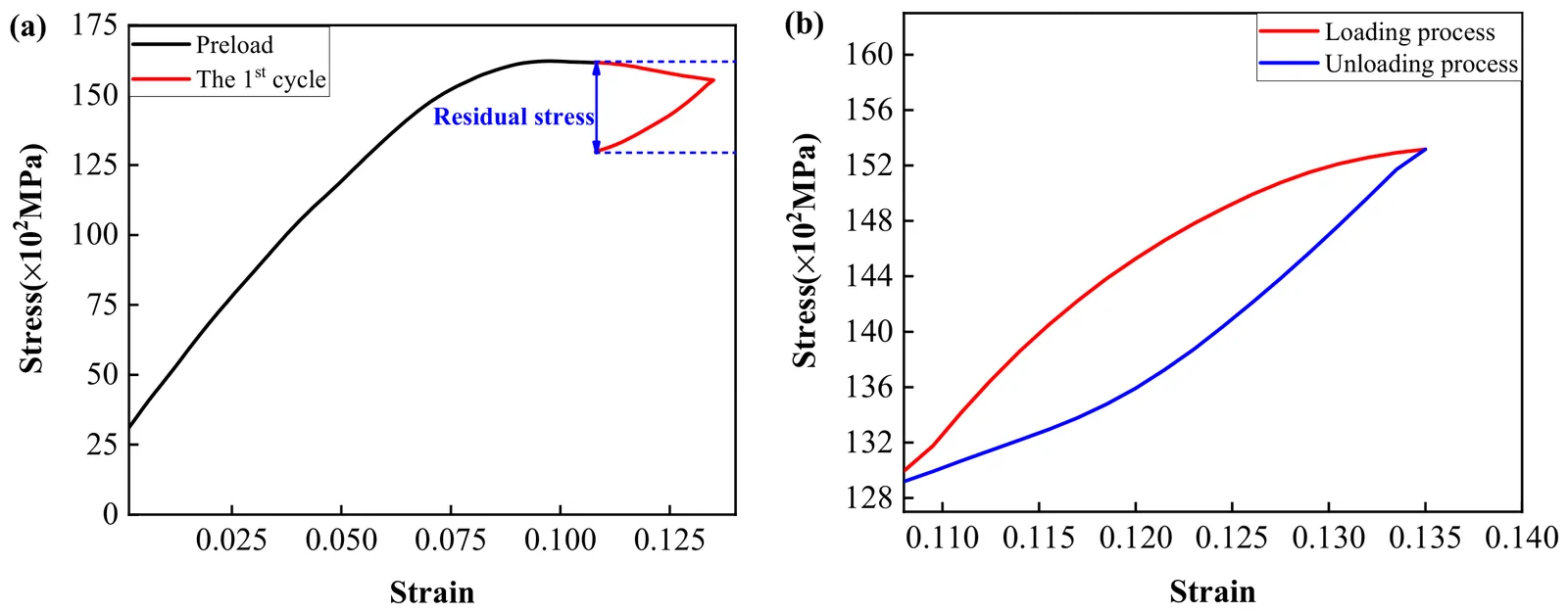
The stress–strain curves during the preload, first, and second successive cyclic loading processes. (**a**) The preload and first cycle processes; (**b**) the second cycle.

The stress–strain curves during the preload and four successive cyclic loading processes are shown in Figure 2 and Figure S2. Figure 2a presents the stress–strain curves of the preload and the first cycle. During the loading process of the first cycle, stress decreased with increasing strain. Then, stress decreased with declining strain during the unloading process. The residual stress of the first cycle was determined to be 31.7 × 10^2^ MPa. Figure 2b and Figure S2a,b indicate that the loading and unloading stress–strain curves of the second, third, and fourth cycles formed a convex lens shape. From Figure 2b, in the loading process of the second cycle, stress showed an increasing trend with increasing strain. Stress presented a decreasing trend with decreasing strain in the unloading process. The stress–strain curves of the third and fourth cycles depicted a similar variation than the second cycle, as shown in Figure S2a,b. During the second cycle, the residual stress was estimated to be zero. During the third cycle, the stress at the unloading ending point was approximately 78 MPa higher than at the loading starting point. During the fourth cycle, the residual stress was estimated to be zero, again.

**Figure 3:**
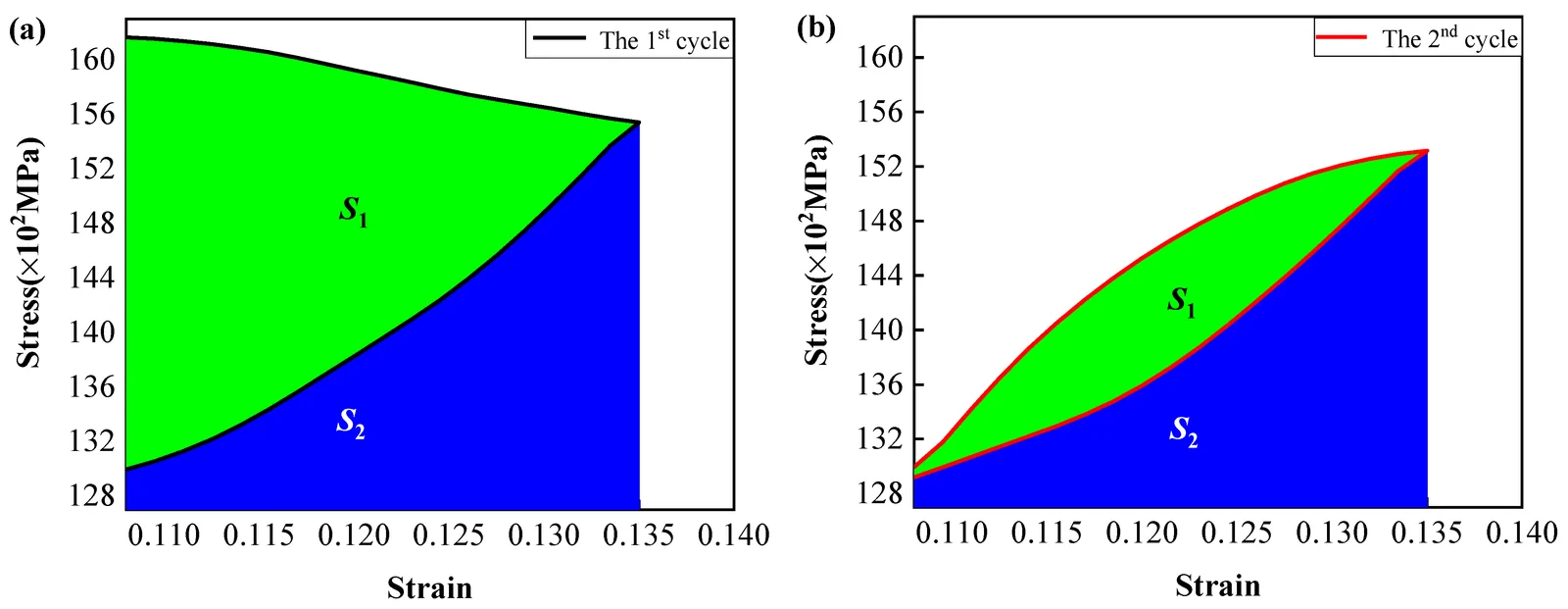
The energy dissipation of the Fe14.6Ni(at%) alloy during the first and second successive cyclic loading processes. (**a**) The first cycle; (**b**) the second cycle.

In addition, Figure 3 and Figure S3 demonstrate the energy dissipation of the Fe14.6Ni(at%) alloy during the four successive cyclic loading processes. *S*_1_ was the area between the loading and the unloading stress–strain curves. From the previous investigation [44], it was known that *S*_1_ represented the energy dissipation during the four successive cyclic loading processes. *S*_2_ was the area between the unloading stress–strain curve and the strain axis, representing the work during the unloading process. The sum of *S*_1_ and *S*_2_ was the work during the loading process. Therefore, the dissipation factor (*D_f_*) was defined as the following formula:

(2) $$D_{f}=\frac{S_{1}}{S_{1}+S_{2}}$$

**Figure 4:**
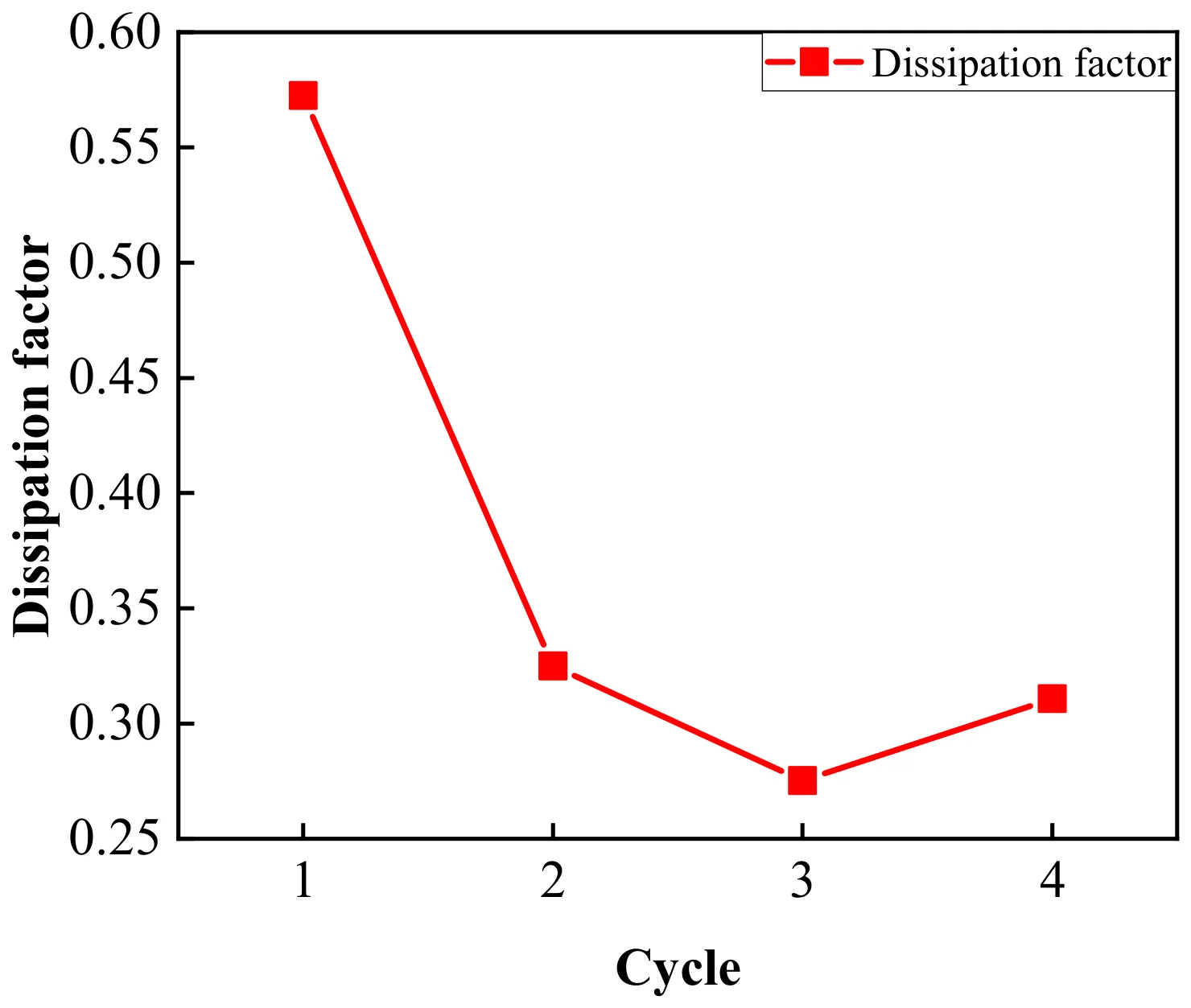
The trend of the *D_f_* during the four cycles.

The trend of the *D_f_* during the four cycles is shown in Figure 4. It can be seen that the *D_f_* decreased steeply after the first cycle and remained around 0.30 during the next three cycles, which shows a small energy dissipation effect [44].

**Figure 5:**
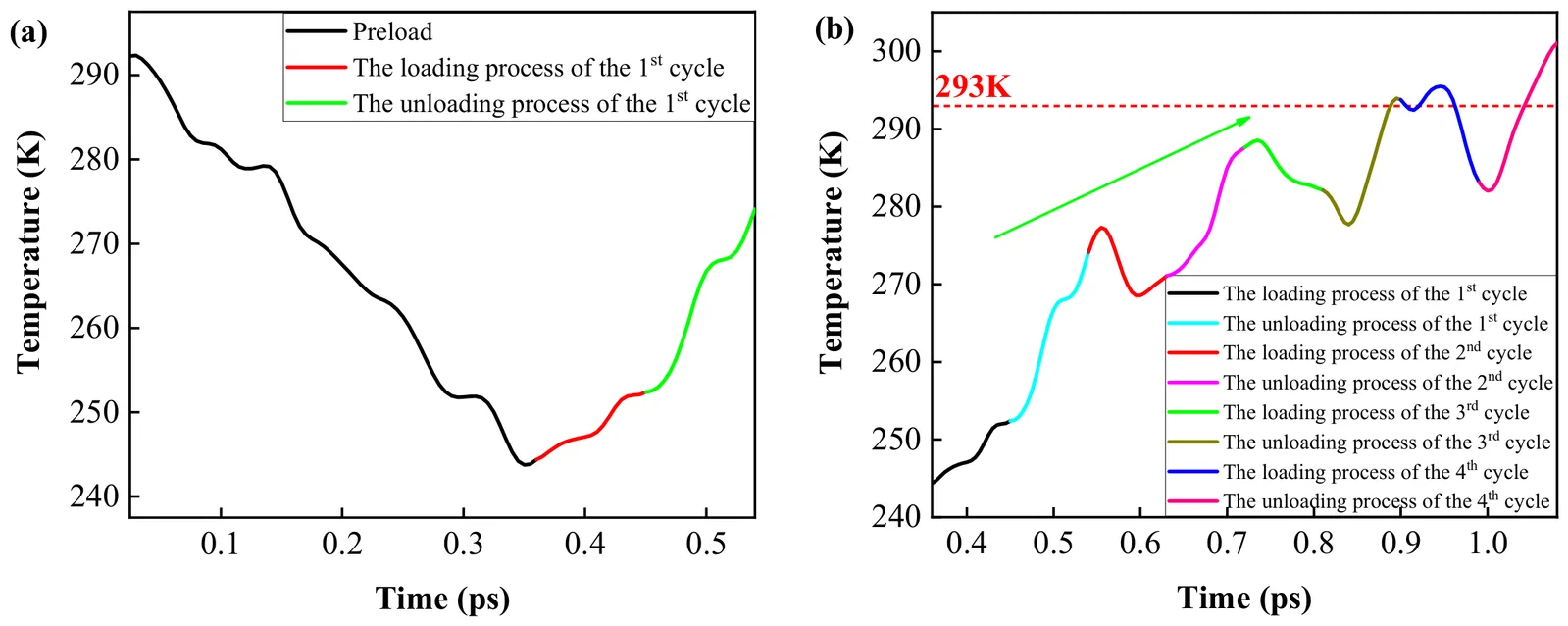
The temperature trends during the preload and four successive cyclic loading processes. (**a**) The preload process and the first cycle; (**b**) the loading processes.

Figure 5 presents the temperature trend during the preload and four successive cyclic loading processes. Figure 5a shows that temperature presented a downward trend during the preload process. During the first cycle process, the temperature showed an upward trend, fluctuating both in the loading and unloading processes. Figure 5b pictures the temperature trend during the four successive cyclic loading processes. As illustrated in Figure 5b, the temperature first increased, then dropped, then rose again with increasing strain during the loading process of the second cycle. The rising trend lasted until the unloading process of the second cycle was completed. For the third cycle, the temperature rose first and then declined during the loading process and an inverse trend can be observed during the unloading process. When the cycle continued onto the fourth cycle, a first dropping, then rising, and finally decreasing trend was depicted in the loading process and an inverse trend was presented in the unloading process.

The temperature increased up to 293 K during the first cycle, the second cycle, and the loading process of the third cycle, as shown in Figure 5b. Even if the partial curves of the unloading process of the third cycle and the fourth cycle crossed the 293 K temperature line, it was difficult to determine the value of the ∆T_adi_ due to the complex variation in temperature. Furthermore, as the green arrow illustrates in Figure 5b, the temperature increased, fluctuating with increasing cycles. This behavior led to the failure of the eCEs due to the high temperatures [31,32].

**Figure 6:**
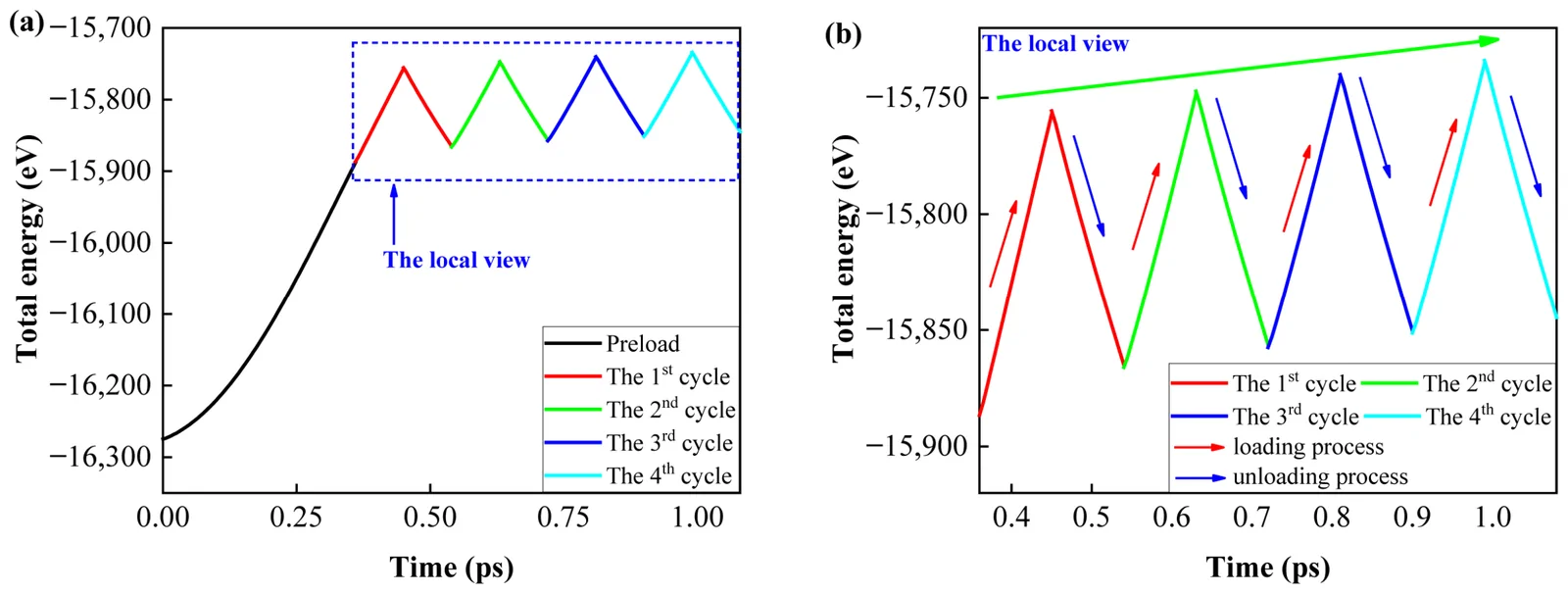
The total energy trends during the preload and four-time successive cyclic loading processes. (**a**) The global view; (**b**) the local view.

Figure 6 shows the total energy trends during the preload and four successive cyclic loading processes. From Figure 6a, it can be seen that the total energy increased during the preload process. The red and blue arrows in Figure 6b depict the total energy trend of the loading and unloading processes in the next three cycles, respectively. It can be seen that the total energy increased during the loading processes and decreased during the unloading processes. As the green arrow indicates in Figure 6b, the total energy presented an increasing standard triangular wave with increasing cycles, which finally caused the energy of the Fe14.6Ni(at%) alloy overload to fail.

**Figure 7:**
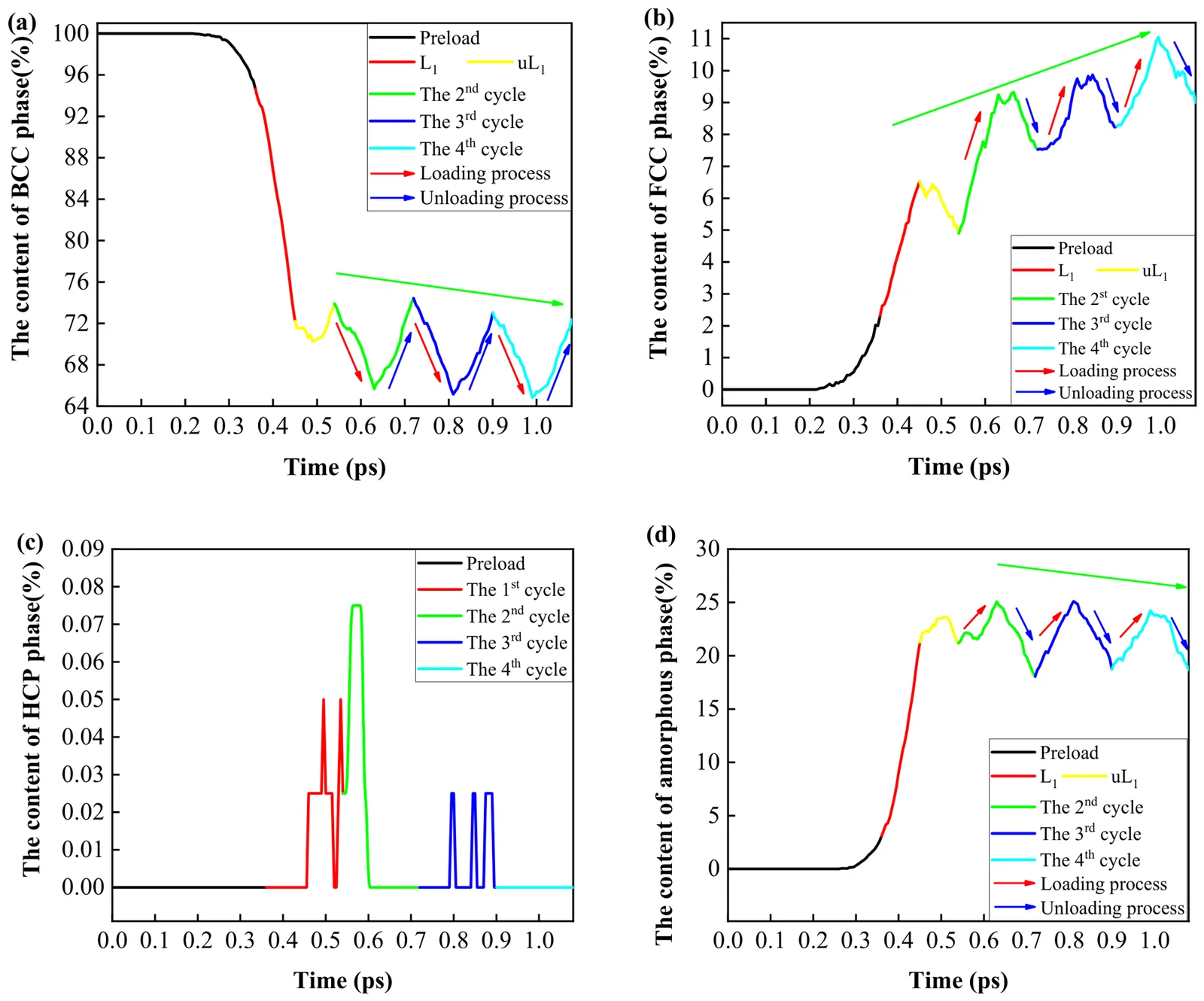
The phase contents evolution trends during the preload and four-time successive cyclic loading processes. (**a**) BCC phase; (**b**) FCC phase; (**c**) HCP phase; (**d**) transition amorphous phase.

The phase contents evolution trends during the preload and four-time successive cyclic loading processes are depicted in Figure 7. In Figure 7, L_1_ and uL_1_ represent the loading and unloading processes of the first cycle, respectively. Figure 7a shows the variation in BCC phase content during the preload and four successive cyclic loading processes. As Figure 7a demonstrates, the BCC phase showed first a constant and then a decreasing trend during the preload process [33]. During the loading process of the first cycle, the content of the BCC phase displayed a steeply declining trend. In the unloading process of the first cycle, the BCC phase presented a first decreasing and then increasing trend. The red and blue arrows in Figure 7a depict the trend of the content of the BCC phase for the loading and unloading processes in the next three cycles, respectively. It was shown that the content of the BCC phase decreased during the loading process and increased during the unloading process. In addition, in the next three cycles, as the green arrow indicates in Figure 7a, the content of the BCC phase presented a declining triangular wave with increasing cycles, causing the consumption of the content of the BCC phase and causing functional failure of the Fe14.6Ni(at%) alloy.

Figure 7b illustrates the trend of the FCC phase content during the preload and four-time successive cyclic loading processes. As Figure 7b presents, in contrast to the trend of the BCC phase content, the content of the FCC phase remained, at first, at zero and then displayed an increasing trend during the preload process [33]. During the first cycle, the content of the FCC phase presented a rapidly rising trend during the loading process and showed a fluctuating downward trend during the unloading process. In the next three cycles, as the red and blue arrows indicate, the content of the FCC phase showed an upward trend during the loading processes and presented a downward trend during the unloading processes. Compared with the varying BCC phase content, during the next three cycles, the content of the FCC phase pictured a rising triangular wave with increasing cycles during the four successive cyclic loading processes, as the green arrow indicates in Figure 7b, contributing to the over-transformation of the FCC phase to functional failure. Figure 7c shows that the content of the HCP phase can be ignored due to the slight changes.

The transition process of the amorphous phase during the preload and four-time successive cyclic loading processes is depicted in Figure 7d. As Figure 7d presents, the content of the transition amorphous phase showed at first a constant zero and then a rising trend during the preload process [33]. During the first cycle, the content of the transition amorphous phase showed a steeply rising trend during the loading process and a first increasing and then decreasing trend in the unloading process, as pictured. In addition, as indicated by the red and blue arrows, the content of the transition amorphous phase showed a similarly evolving trend to that of the FCC phase content during the loading and unloading processes in the next three cycles. After the preload and the first cycle process, the content of the transition amorphous phase presented a declining triangular wave with increasing cycles, which exhausted the transition amorphous phase with the increasing cycles and caused functional destruction of the Fe14.6Ni(at%) alloy.

**Figure 8:**
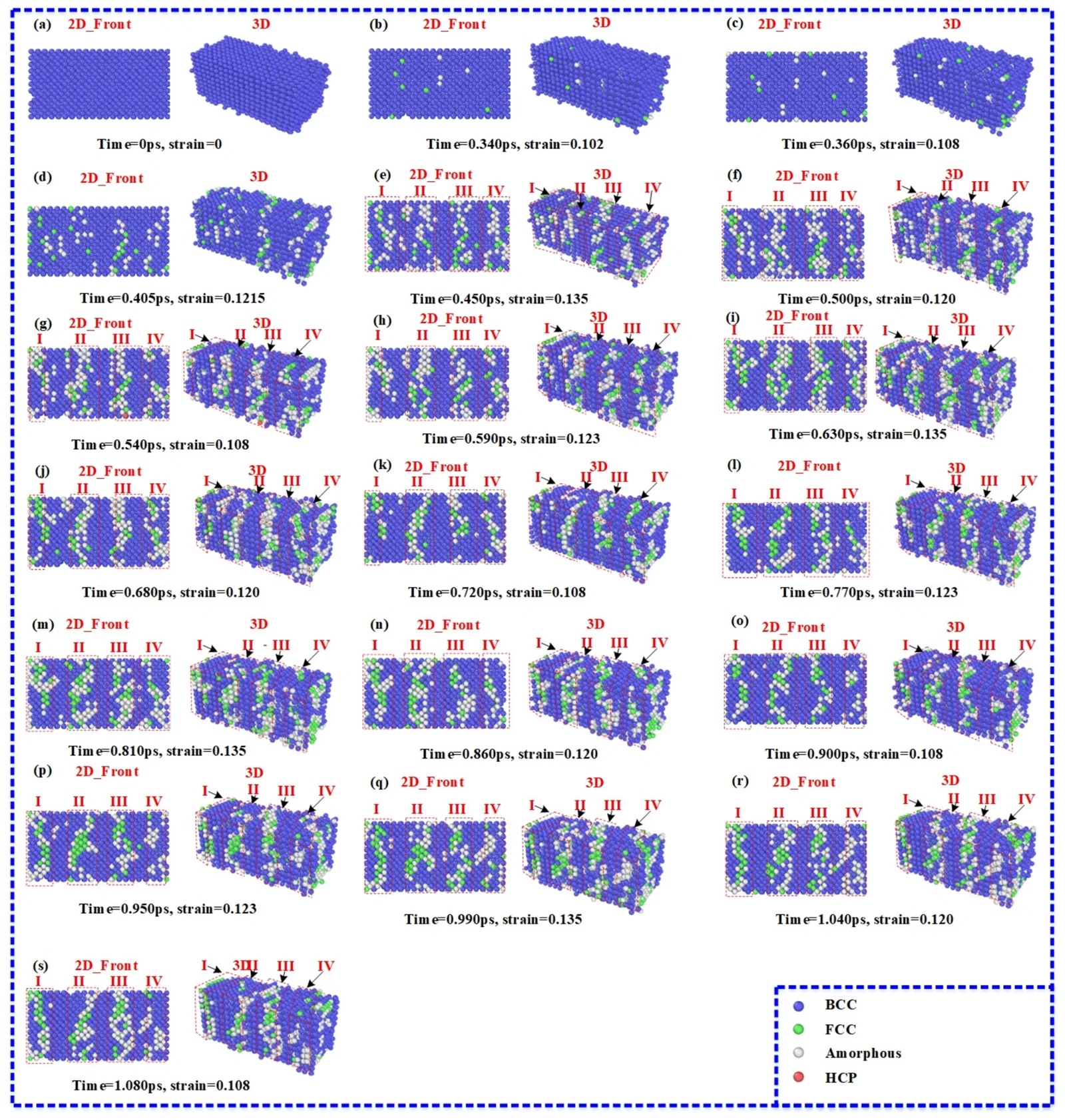
The crystal structure evolution during the preload and four-time successive cyclic loading processes. (**a**) Time = 0 ps; (**b**) time = 0.340 ps; (**c**) time = 0.360 ps; (**d**) time = 0.405 ps; (**e**) time = 0.450 ps; (**f**) time = 0.500 ps; (**g**) time = 0.540 ps; (**h**) time = 0.590 ps; (**i**) time = 0.630 ps; (**j**) time = 0.680 ps; (**k**) time = 0.720 ps; (**l**) time = 0.770 ps; (**m**) time = 0.810 ps; (**n**) time = 0.860 ps; (**o**) time = 0.900 ps; (**p**) time = 0.950 ps; (**q**) time = 0.990 ps; (**r**) time = 1.040 ps; (**s**) time = 1.080 ps.

The crystal structure evolution process during the preload and four successive cyclic loading processes is pictured in Figure 8. Figure 8a–c show the crystal structure evolution during the preload process. From Figure 8a, it can be seen that the Fe14.6Ni(at%) alloy is structured in the BCC phase when time = 0 ps. As the time increased, the crystal structure was excited by external stimuli, which transformed the BCC phase to the FCC phase and the transition amorphous phase. The transformed FCC and transition amorphous phases are distributed randomly in the Fe14.6Ni(at%) alloy, as shown in Figure 8b. As the time reached 0.360 ps, the preload process ended, and the number of transformed atoms in the FCC phase and the transition amorphous phase increased, as illustrated by Figure 8c. Figure 8c–e describes the crystal structure evolution during the loading process of the first cycle. When time = 0.405 ps, the number of transformed atoms continued to rise and presented a random distribution, as shown in Figure 8d. At time = 0.450 ps, shown in Figure 8e, the transformed atoms roughly gathered in area I, area II, area III, and area IV, in which the crystal orientations were (100), (100), (210), and (100), respectively. The crystal orientations are determined by OVITO [35]. Figure 8e–g depict the crystal structure evolution during the unloading process of the first cycle. When the time = 0.500 ps, as described in Figure 8f, the transformed atoms scattered stochastically and were collected in area I, area II, area III, and area IV, in which the crystal orientation was approximately transformed into (100), (210), (210) and (210), respectively. When time = 0.540 ps, as shown in Figure 8g, the crystal structure was similar to the state when time = 0.500 ps, except for the exact distribution of the transformed atoms. The unloading process of the first cycle came to an end and the loading process of the second cycle commenced when time = 0.540 ps.

During the next three processes, the second, the third, and the fourth cycles, the transformed atoms clustered in area I, area II, area III, and area IV regularly with the crystal orientation (100), (210), (210) and (210), respectively. However, the transformed atoms scattered roughly and mutually penetrated the structure, leading to the fixed-structure clusters not forming in the 2D front view or in the 3D perspective, as shown in Figure 8h–s.

In summary, the results showed that the four successive cyclic loading method was not favorable due to the significant stress hysteresis, the chaos, the unstable state of temperature, the consumption of BCC phase, and the disordered crystal structure distribution.

### 3.2. The Investigation of the eCEs Performance Using the Pulse Cyclic Loading Method

In Section 3.1, the successive cyclic loading method was investigated and it was found that the successive cyclic loading method was not favorable for enhancing the eCEs performance [15,18,19,21]. Therefore, the pulse cyclic loading method was proposed by introducing relaxation processes to the cycles to reform the successive cyclic loading method [13].

**Figure 9:**
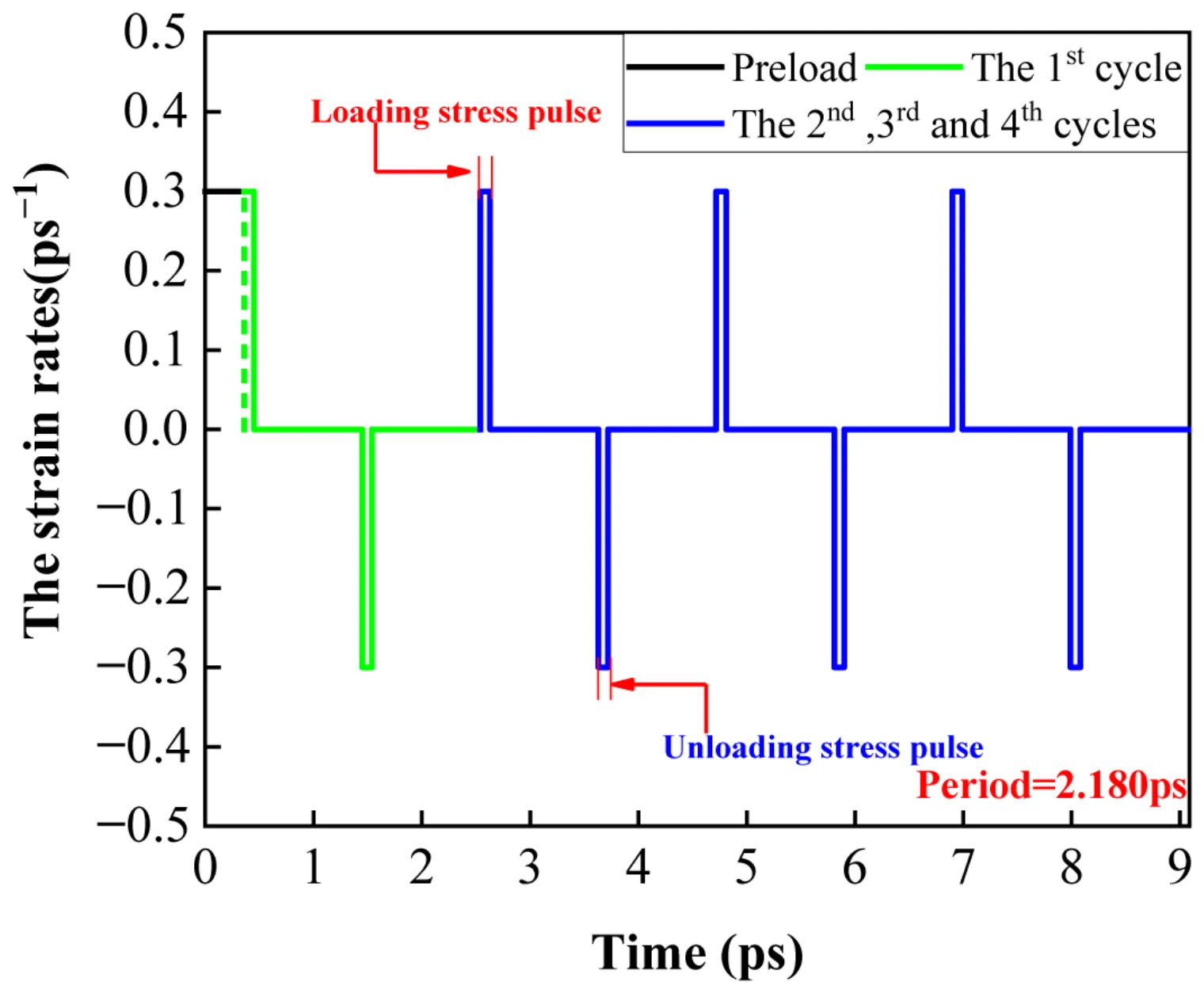
The stress pulses during the preload and four-time pulse cyclic loading processes.

Two relaxation processes followed the loading and unloading process and lasted for 1 ps. The period was 2.180 ps and the stress pulses’ widths during the loading and unloading processes were both 4.128%. The other operations were the same as in Section 3.1. Figure 9 depicts the stress pulses during the preload and four cyclic loading processes.

**Figure 10:**
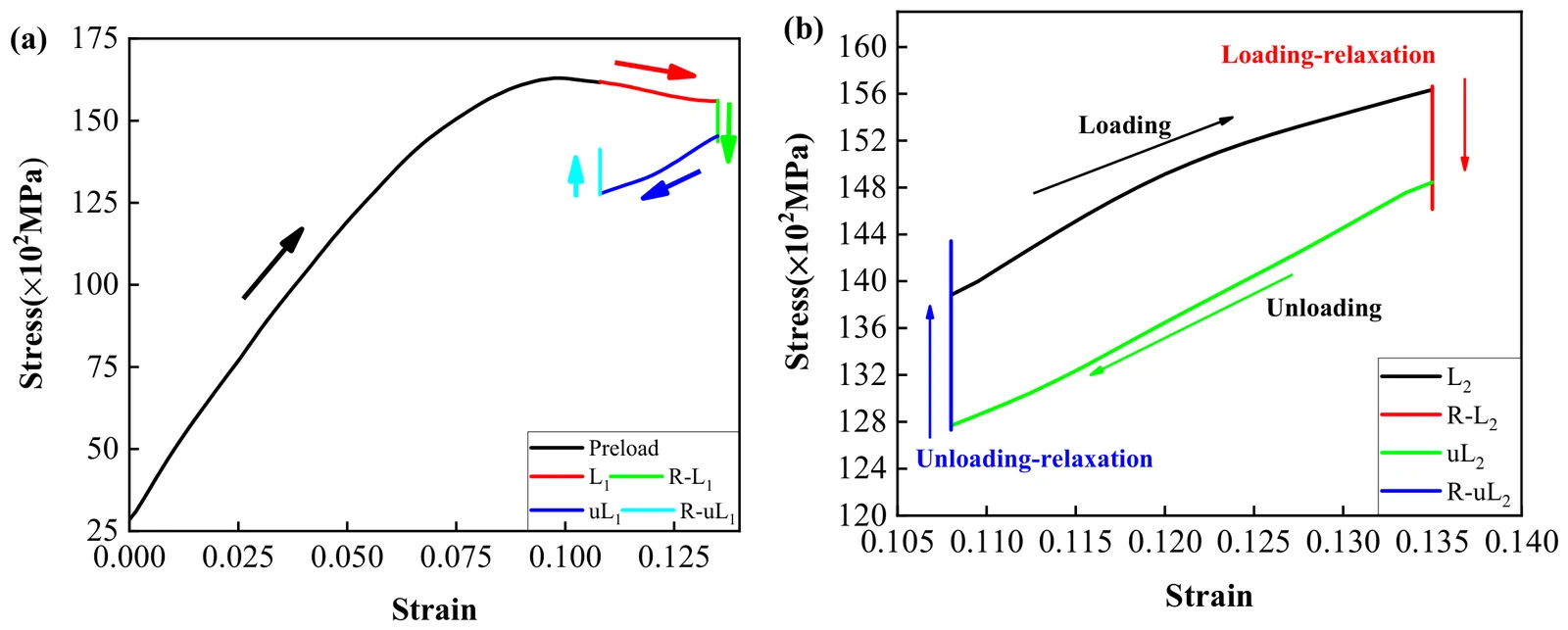
The stress–strain curves during the preload and four-time pulse cyclic loading processes. (**a**) The preload process and the first cycle; (**b**) the second cycle.

The stress–strain curves during the preload and four-time pulse cyclic loading processes are shown in Figure 10 and Figure S4. R-L and R-uL represent the loading–relaxation process and unloading–relaxation process, respectively. Figure 10a presents the stress–strain curves of the preload and the first cycle. The residual stress of the first cycle was determined to be 16.5 × 10^2^ MPa, approximately half of the residual stress of the first cycle during the successive cyclic loading process. Different from the convex lens shape of the successive cyclic loading processes, Figure 10b indicated that the stress–strain curve of the second cycle was a closed loop and presented the shape of a curve-enclosed parallelogram. Similarly to the varying trend of the second cycle in the successive cyclic loading processes, the stress increased with strain during the loading process and decreased with strain during the unloading process. Different from the successive cyclic loading processes, the strain remained at 0.135 and 0.108 during the two relaxation processes. The trend of stress during the second cycle demonstrated that both relaxation processes were necessary to eliminate residual stress. The specific duration of both relaxation processes was crucial. An overlong duration could lead to a low efficiency and an overly short duration might cause large stress hysteresis. A large stress hysteresis would contribute to the stress overload, causing failure of the Fe14.6Ni(at%) alloy structure. During the second cycle, the residual stress was determined to be 80.38 MPa. Similarly to the second cycle, Figure S4a,b depicts the stress–strain curves of the third and the fourth cycles as closed loop and curve-enclosed parallelogram shapes. During the third cycle, the stress at the third unloading ending point was approximately 38.52 MPa lower than at the third loading starting point. During the fourth cycle, the residual stress was 59.85 MPa. Different from the residual stress of the first cycle during the preload and successive cyclic processes, the residual stress existed in every cycle and remained at a low level.

**Figure 11:**
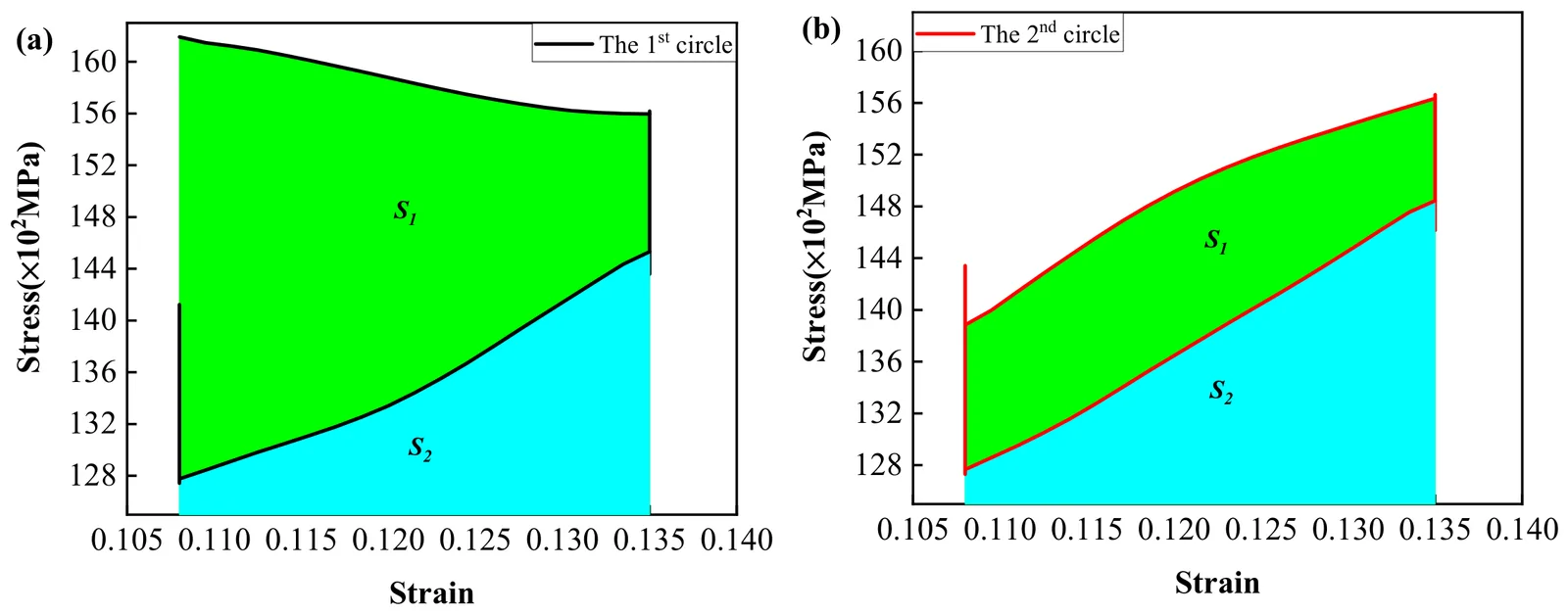
The energy dissipation of the Fe14.6Ni(at%) alloy during the first and the second pulse cyclic loading processes. (**a**) The first cycle; (**b**) the second cycle.

**Figure 12:**
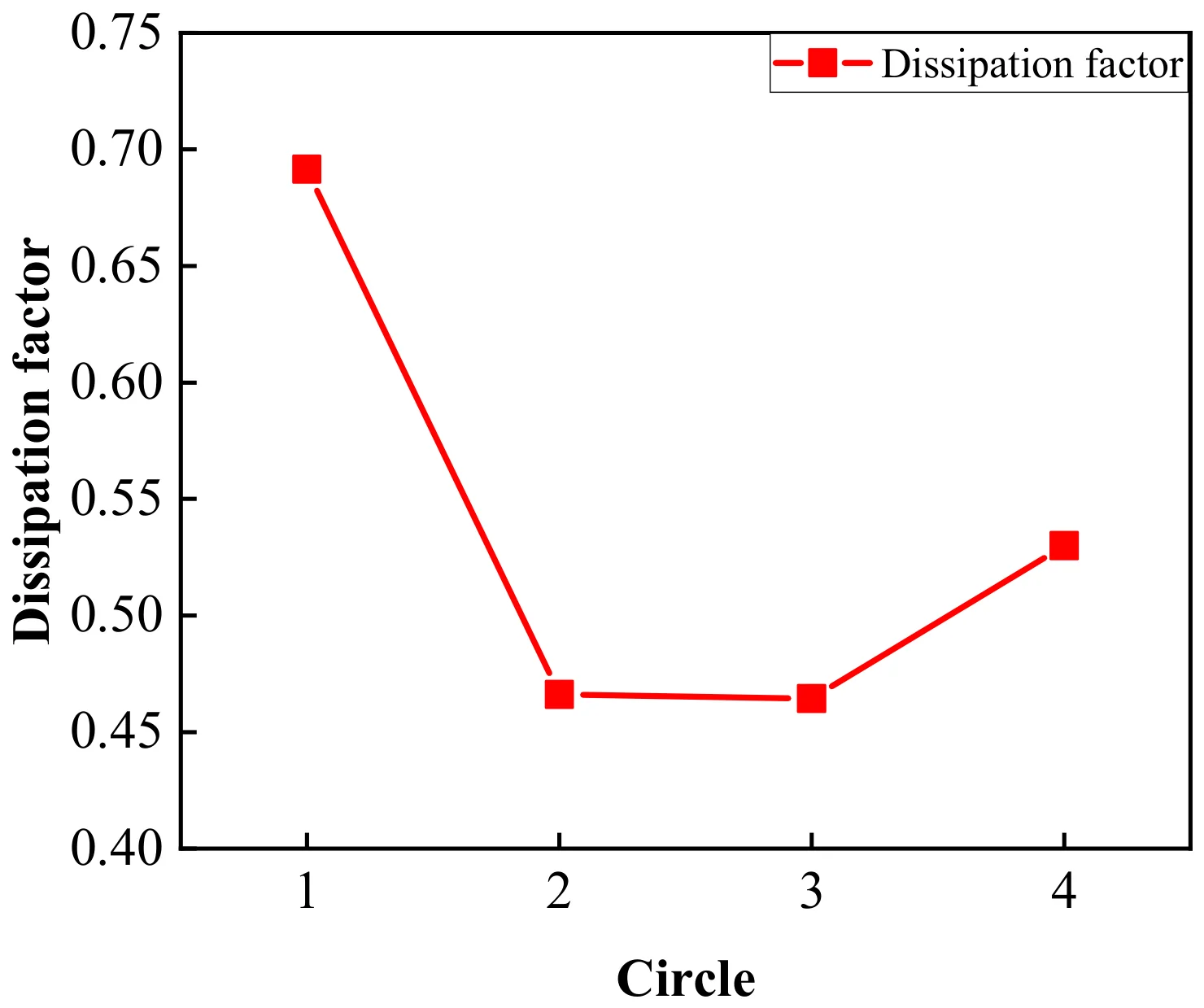
The trend of the Df during the four cycles.

**Figure 13:**
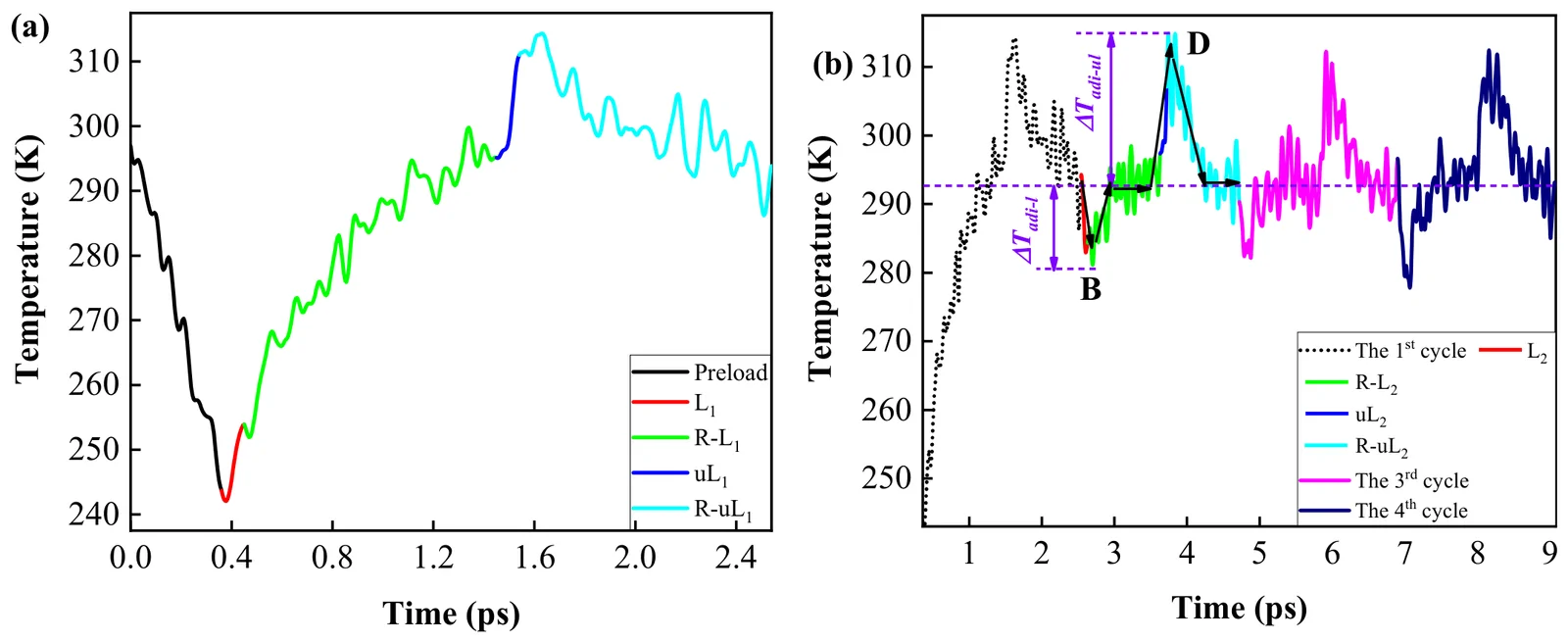
The temperature trends during the preload and four-time pulse cyclic loading processes. (**a**) The preload process and the first cycle; (**b**) the four-time pulse cyclic loading processes.

Figure 11 and Figure S5 illustrate the energy dissipation of the Fe14.6Ni(at%) alloy during the four-time pulse cyclic loading processes. The *D_f_* can be determined from Equation (2). Figure 12 presents the trend of the *D_f_* during the four cycles. In Figure 12, it can be seen that the *D_f_* decreased sharply after the first cycle and remained around 0.5 with increasing cycles; a value 1.67 times bigger than that of the successive cyclic processes, due to the relaxation process. Compared with successive cyclic processes, indeed, pulse cyclic loading processes lead to more energy dissipation. However, more energy dissipation creates a refrigeration process of a more promising stability and a longer thermal fatigue life than with successive cyclic processes. Figure 13 depicts the temperature trends during the preload and four-time pulse cyclic loading processes. In Figure 13a, the temperature presented a downward trend during the preload process. In the first cycle, the temperature showed a fluctuating upward trend in the loading process, the loading–relaxation process, and the unloading process. The temperature showed a fluctuating decreasing trend in the unloading–relaxation process.

Figure 13b depicts the temperature trend during the four-time pulse cyclic loading processes. As the arrows show, in Figure 13b, temperature showed a steadily decreasing trend during the loading process of the second cycle. During the loading–relaxation process, the temperature showed, first, a fluctuating increase and then a stable trend (around 293 K). In the unloading process, the temperature rose sharply. During the unloading–relaxation process, the temperature fluctuation first dropped and then remained stable (around 293 K). The trend of the temperature during the loading–relaxation and unloading–relaxation processes demonstrated that both the relaxation processes were important for energy absorption and release to ensure temperature recovery to room temperature. The duration of the relaxation processes needed to be set reasonably to prevent the low cooling efficiency caused by an extended duration and the overcooling or overheating of the Fe14.6Ni(at%) alloy caused by a shorter duration. During the second cycle, the ∆T_adi_ values during the loading and unloading process were determined to be −12 K and 22 K, respectively. In the third and fourth cycles, the temperature showed a similar trend to the second cycle. The ∆T_adi_ values in the third and fourth cycles were determined to be −11 K/19 K and −16 K/19 K during loading and unloading processes, respectively. Notably, as shown in Figure 13b, the temperature varied both lower and higher than 293 K during the four-time loading and unloading processes, presenting a universal trend and reliable values of ∆T_adi_. This is vital not only for stable eCEs performance but also for potential practical applications.

**Figure 14:**
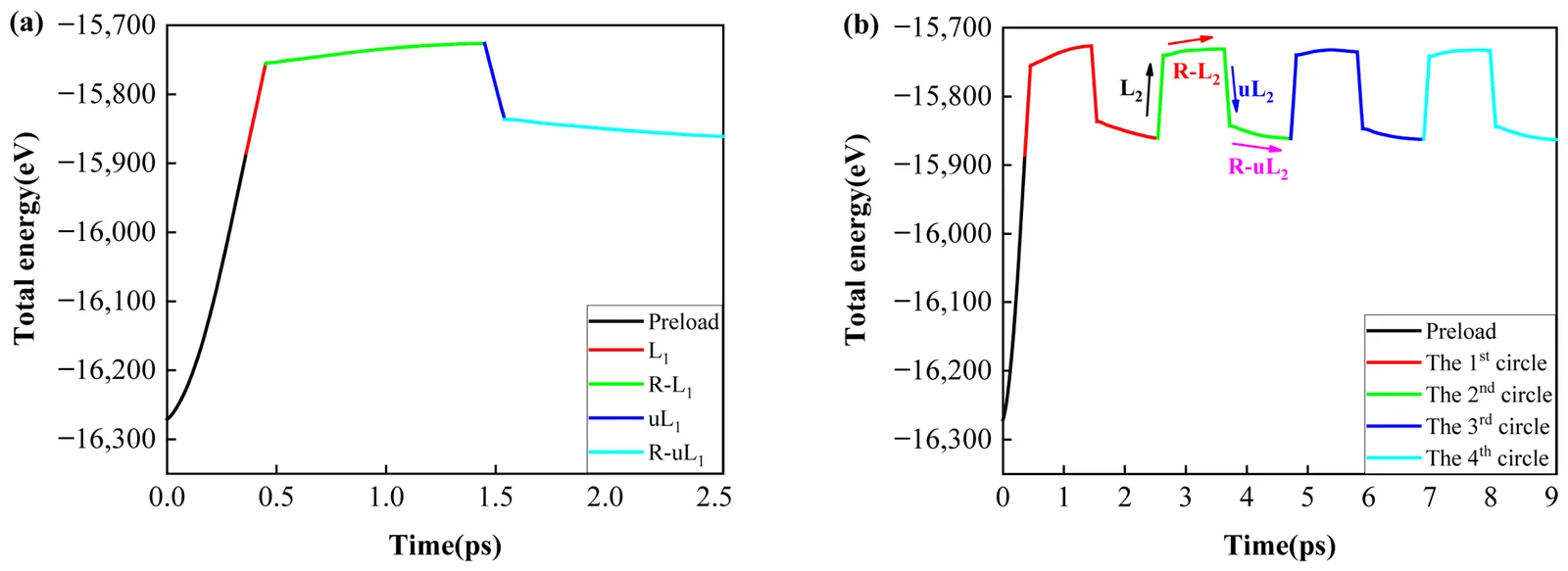
The total energy trends during the preload and the four-time pulse cyclic loading processes. (**a**) The preload process and the first cycle; (**b**) the four-time pulse cyclic loading processes.

Figure 14 shows the total energy trends during the preload and the four-time pulse cyclic loading processes. From Figure 14a, it can be seen that the total energy increased during the preload process. The total energy presented a sharply increasing trend during the loading process of the first cycle. During the loading–relaxation process of the first cycle, the total energy showed an extremely slow-rising trend. The total energy dropped steeply in the unloading process of the first cycle, while the total energy declined smoothly during the unloading–relaxation process. As shown in Figure 14b, the total energy showed a similar evolution process to the first cycle during the next three cycles. The trend during both relaxation processes demonstrated that the relaxation processes played an important role in recovering the total energy to an energy-minimized state. Notably, the duration of the relaxation process should be reasonable to avoid the low efficiency caused by a long duration or a functional failure due to a short duration.

**Figure 15:**
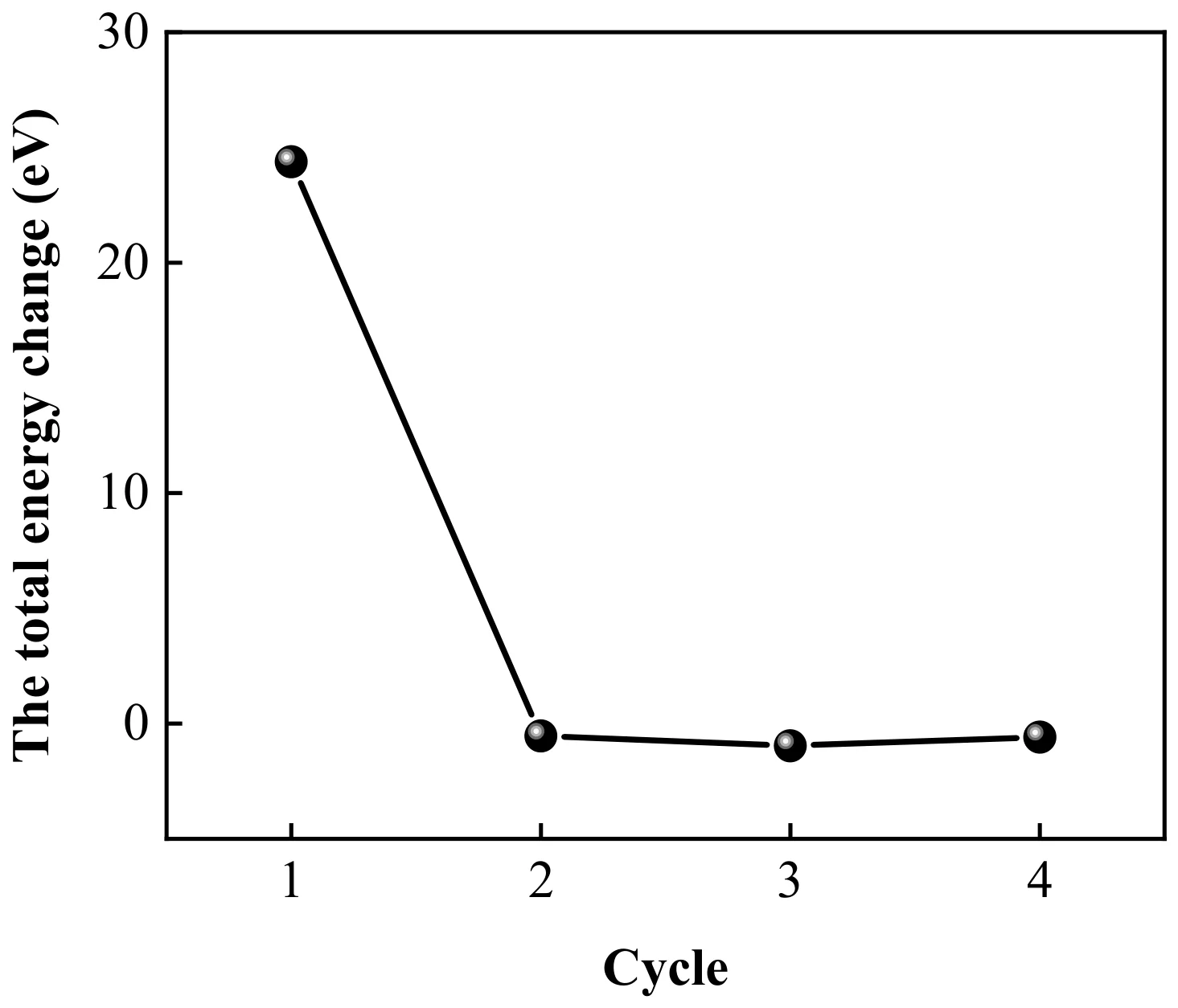
The trend of the total energy change during the four cycles.

The total energy changes were also determined during the four cycles. The total energy change was defined as the difference between the ending and the starting total energy in one cycle, to describe the stability of the cycle from an energy aspect. The trend of the total energy change during the four cycles is pictured in Figure 15. It can be seen that the total energy change, first, dropped and then remained around zero, which illustrates that the total energy evolved steadily after the first cycle.

**Figure 16:**
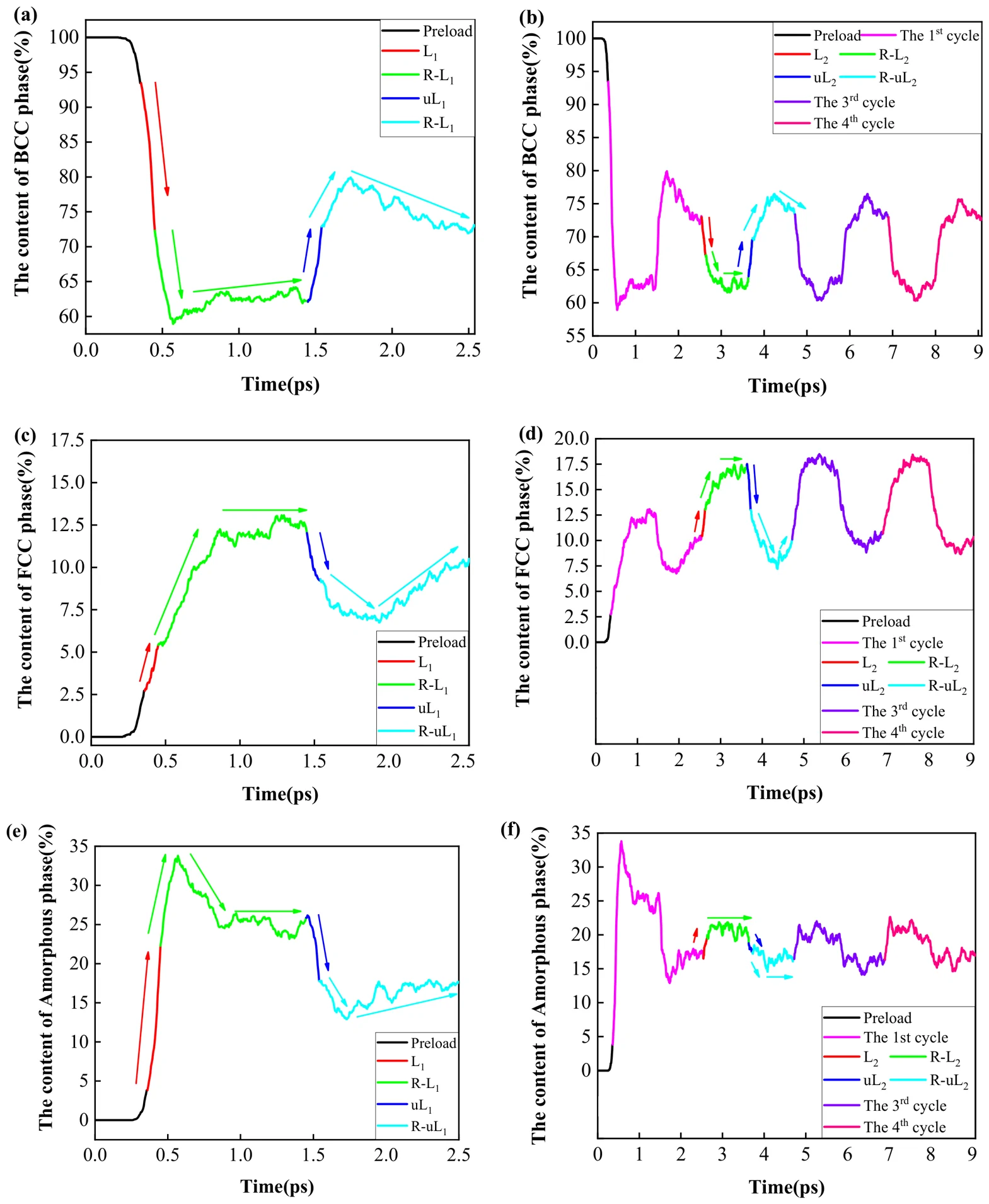
The phase content evolution during the preload and four-time pulse cyclic loading processes. (**a**,**b**) BCC phase; (**c**,**d**) FCC phase; (**e**,**f**) amorphous phase.

The phase contents evolution during the preload and four-time pulse cyclic loading processes is presented in Figure 16. Figure 16a,b show the trends of the BCC phase content during the preload and pulse cyclic processes. As Figure 16a demonstrates, the BCC phase showed, first, a constant, and then a declining trend during the preload process [33]. During the loading process of the first cycle, the BCC phase showed a steeply declining trend. In the loading–relaxation process of the first cycle, the BCC phase presented a first decreasing and then fluctuating increasing trend. During the unloading process of the first cycle, the BCC phase increased sharply. In the unloading–relaxation process of the first cycle, the BCC phase rose, first, and then decreased with fluctuation. Figure 16b depicts the trend of the BCC phase content during the four-time pulse cyclic loading processes. During the next three cycles, taking the second cycle as an instance, the BCC phase depicted a similar trend as the first cycle of the BCC phase. The content of the BCC phase during the third and the fourth cycles presented a nearly identical trend to the second cycle, which proved that the BCC phase transformed regularly after the second cycle.

The trends of the FCC phase content during the preload and pulse cyclic loading processes are presented in Figure 16c,d. Figure 16c demonstrates that the FCC phase depicted an initially constant and then an increasing trend during the preload process [33]. The FCC phase increased steeply during the loading process of the first cycle. In the loading–relaxation process of the first cycle, the FCC phase increased first and then remained stable with fluctuation. During the unloading process of the first cycle, the FCC phase decreased sharply. In the unloading–relaxation process of the first cycle, the FCC phase showed a downward and then upward trend with fluctuation. Figure 16d describes the trend of the FCC phase during the four-time pulse cyclic loading processes. During the next three cycles, taking the second cycle as an example, the FCC phase depicted a similar trend to the first cycle, of the FCC phase. The FCC phase of the third and fourth cycles presented nearly identical trends to the second cycle, illustrating that the FCC phase transformed regularly after the second cycle.

Figure 16e,f describe the trends of the transition amorphous phase content during the preload and pulse cyclic loading processes. From Figure 16e, it can be seen that the transition amorphous phase presented an initially constant and then rising trend during the preload process [33]. During the loading process of the first cycle, the transition amorphous phase depicted a steeply rising trend. In the loading–relaxation process of the first cycle, the transition amorphous phase presented an increasing, then decreasing, and finally stable trend. During the unloading process of the first cycle, the transition amorphous phase dropped steeply. In the unloading–relaxation process of the first cycle, the transition amorphous phase declined first and then increased with fluctuation. Figure 16e depicts the trend of the transition amorphous phase content during the four-time pulse cyclic loading processes. As shown in Figure 16f, the transition amorphous phase content rose steeply during the loading process of the second cycle. Unlike the first cycle, the transition amorphous phase remained stable with fluctuation during the loading–relaxation process of the second cycle. Similarly to the first cycle, the transition amorphous phase presented a sharp decreasing trend during the unloading process of the second cycle. In the unloading–relaxation process of the second cycle, the transition amorphous phase dropped first and then remained steady with fluctuation, which differed from the first cycle. During the next two cycles, the transition amorphous phase of the third and the fourth cycles showed nearly identical trends to the second cycle, demonstrating that the transition amorphous phase transformed regularly after the second cycle.

In summary, the transformation from the BCC phase to the FCC and amorphous phase gradually became stable after the second cycle. From the above analysis, the conclusion can be drawn that the relaxation processes were required in order to optimize the BCC, FCC, and transition amorphous phases into favorable contents. The duration of the relaxation process is postulated to be appropriate to prevent low efficiency and the consumption of the BCC phase induced by a long duration, leading to the functional failure of the Fe14.6Ni(at%) alloy.

**Figure 17:**
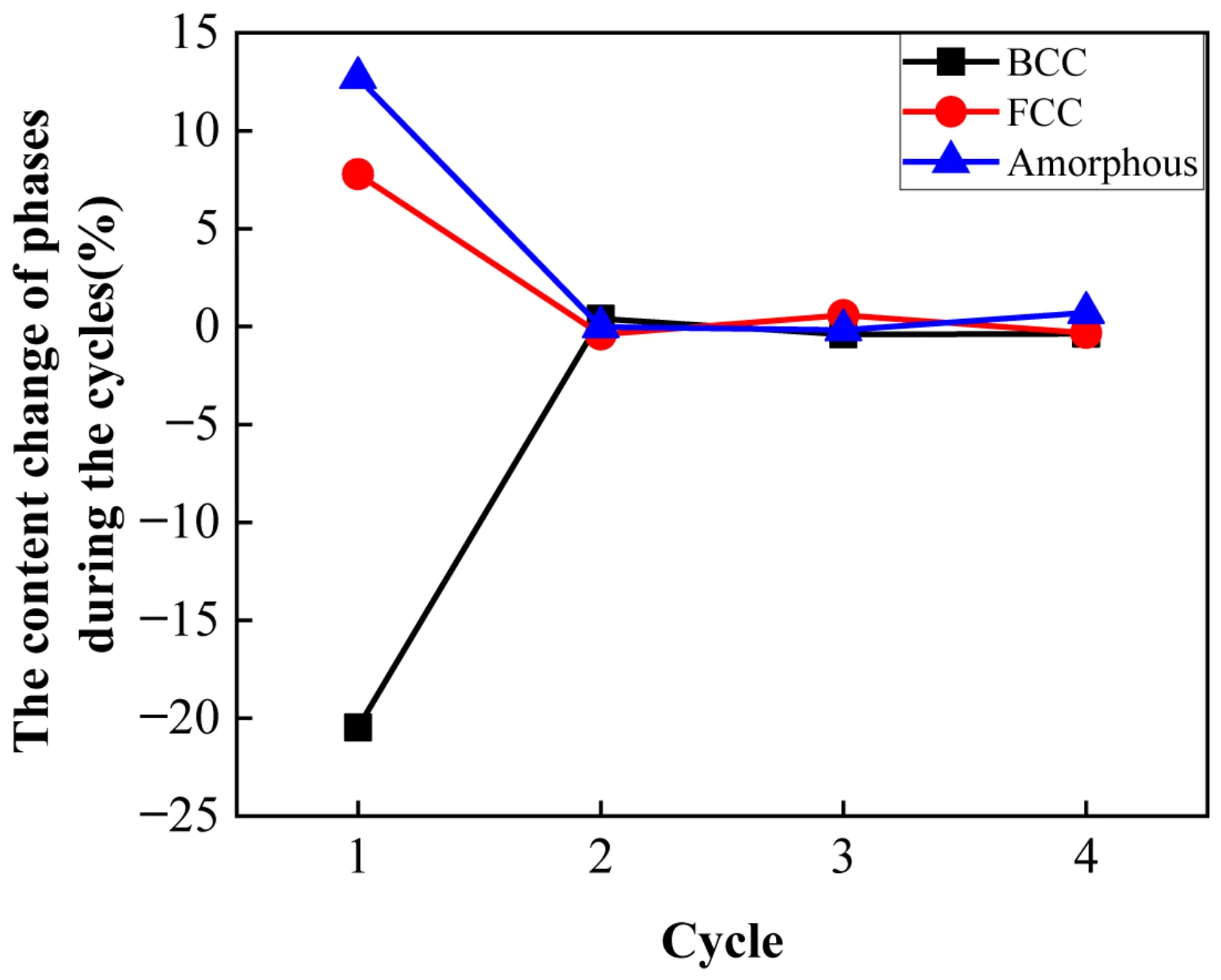
The content changes in the BCC, FCC and amorphous phases during the four cycles.

The phase content change was defined as the difference between the ending and the starting phase content of one cycle, to describe the stability of the cycle in a phase content aspect. The content changes in BCC, FCC, and amorphous phases during the four cycles are depicted in Figure 17. Figure 17 shows that the content changes in BCC and amorphous phases increased first and remained at about zero. The content changes in the FCC and amorphous phases decreased to zero. This demonstrates that the second, third, and fourth cycles evolved in a steady process.

**Figure 18:**
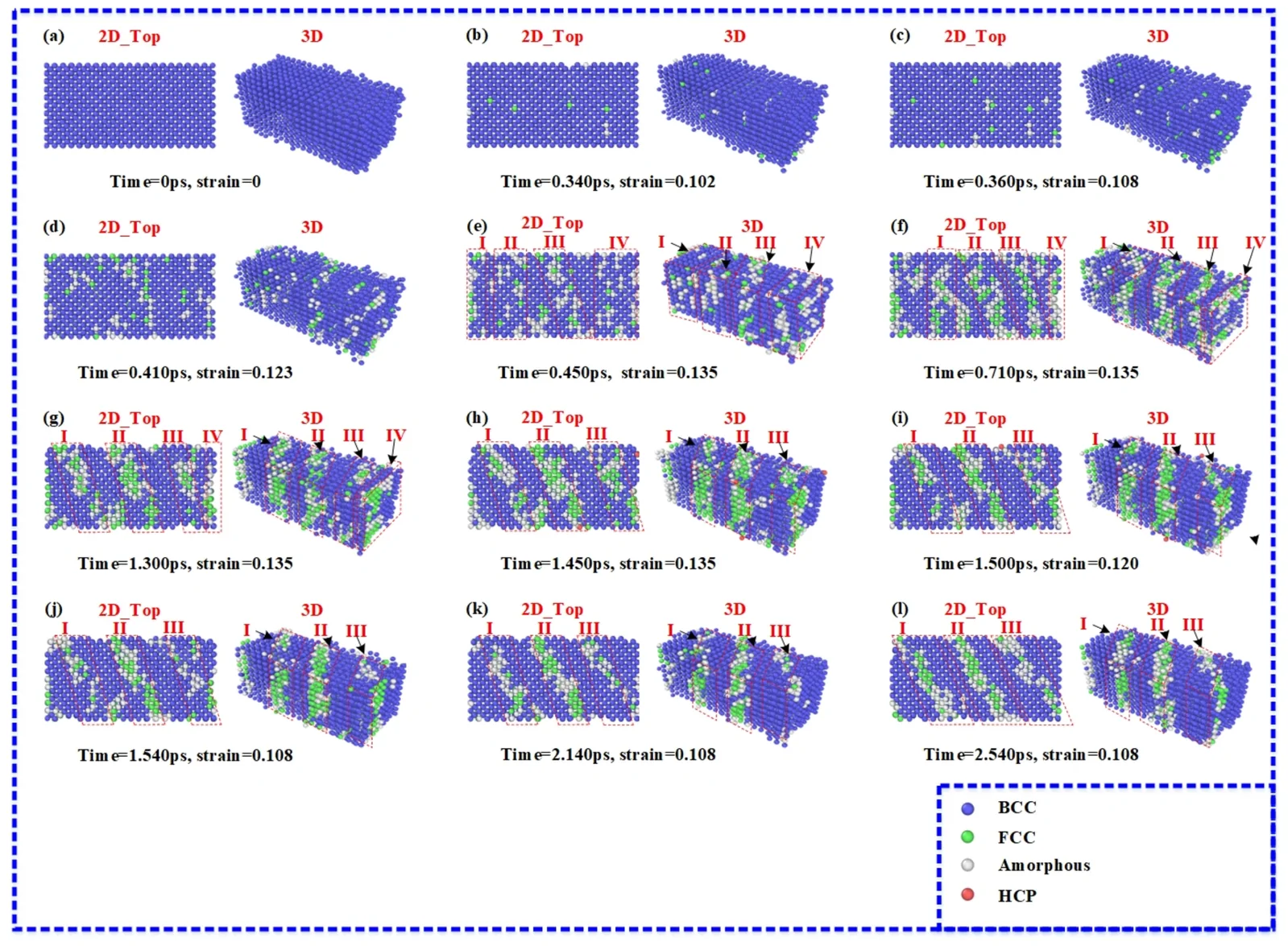
The crystal structure evolution during the preload and the first cycle processes. (**a**) Time = 0 ps; (**b**) time = 0.340 ps; (**c**) time = 0.360 ps; (**d**) time = 0.410 ps; (**e**) time = 0.450 ps; (**f**) time = 0.710 ps; (**g**) time = 1.300 ps; (**h**) time = 1.450 ps; (**i**) time = 1.500 ps; (**j**) time = 1.540 ps; (**k**) time = 2.140 ps; (**l**) time = 2.540 ps.

The crystal structure evolutions during the preload and the first cycle processes are shown in Figure 18. As shown in Figure 18a–c, the crystal structure evolution of the preload process during the pulse cyclic loading processes was the same as the successive cyclic loading processes. Similarly, as described in Figure 8c–e, during the loading process of the first cycle, the transformed atoms were roughly assembled in area I, area II, area III, and area IV. The four areas of crystal orientation were (100), (100), (210), and (100), in order. The loading process of the first cycle was the formation stage of the transition bonds. At time = 0.450 ps, the loading process of the first cycle was completed and the first loading–relaxation process started. Figure 18e–h presents the loading–relaxation process. As pictured in Figure 18f, when the time increased from 0.450 ps to 0.710 ps, area II advanced to transform from a (1 0 0) to a (2 1 0) crystal orientation and roughly formed an independent transition bond. When time increased to 1.300 ps, shown in Figure 18g, the area I followed and transformed from a (1 0 0) to a (2 1 0) crystal orientation and became roughly shaped into an independent transition bond, as well. When the time increased to 1.450 ps, area IV merged with area III and area III, as the last area, converted from a (1 0 0) to a (2 1 0) crystal orientation, forming an independent transition bond. Thus, the first loading–relaxation process performed the role of forming and organising the transition bonds.

When the time reached 1.450 ps, the first loading–relaxation process ended and the unloading process of the first cycle began. The unloading process of the first cycle is presented in Figure 18h–j. Figure 18i,j describes the crystal structure states when the time was 1.500 ps and 1.540 ps, respectively. These two crystal structure states illustrate that area IV totally merged with area III and the three transition bonds with a (2 1 0) crystal orientation were finally formed. At a time of 1.540 ps, the unloading process of the first cycle finished and the first unloading–relaxation process commenced. Figure 18j–l show the first unloading–relaxation process. The crystal structure states of 2.140 ps and 2.540 ps are shown in Figure 18k and Figure 18l, respectively. These two crystal structure states demonstrate that the transformed atoms of FCC and transition amorphous phase adjusted their location, contributing to the official formation of the “‘BCC’-‘transition amorphous’-‘FCC’-‘transition amorphous’-‘BCC’” five-layer structure transition bonds with a (2 1 0) crystal orientation. These phenomena demonstrate that the relaxation processes were necessary to form the ordered transition bonds. Moreover, the duration should be appropriate to prevent the low efficiency caused by a long duration and the incomplete self-healing of the transition bonds that decrease the eCEs performance of the Fe14.6Ni(at%) alloy.

**Figure 19:**
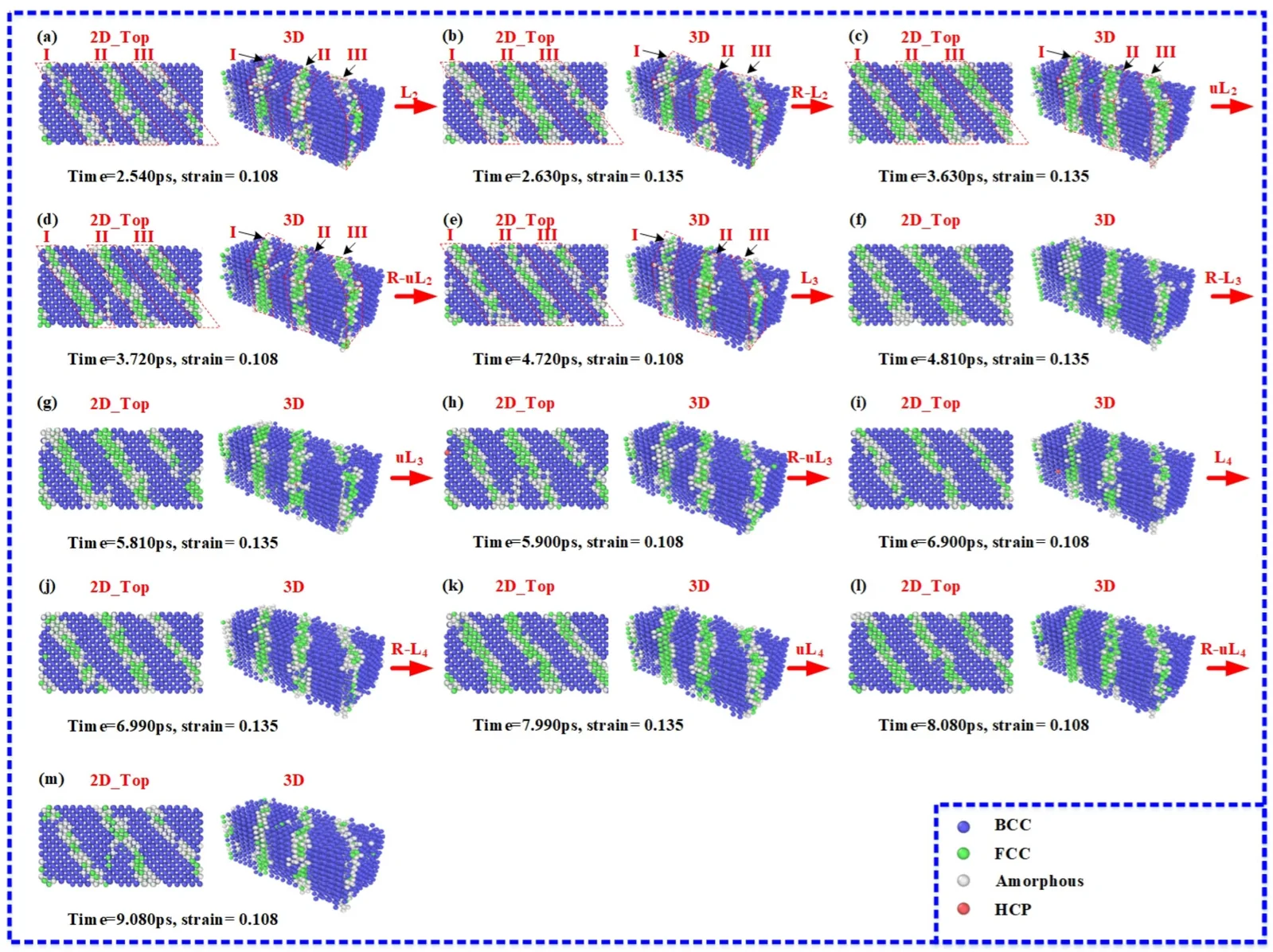
The crystal structure evolution during the second, the third, and the fourth cycles. (**a**) Time = 2.540 ps; (**b**) time = 2.630 ps; (**c**) time = 3.630 ps; (**d**) time = 3.720 ps; (**e**) time = 4.720 ps; (**f**) time = 4.810 ps; (**g**) time = 5.810 ps; (**h**) time = 5.900 ps; (**i**) time = 6.900 ps; (**j**) time = 6.990 ps; (**k**) time = 7.990 ps; (**l**) time = 8.080 ps; (**m**) time = 9.080 ps.

Figure 19 describes the crystal structural evolution during the second, third, and fourth cycles. Figure 19a, Figure 19e, and Figure 19i are the initial state of the loading processes of the second, the third, and the fourth cycles, respectively. The initial state of the loading–relaxation processes of the second, third, and fourth cycles is depicted in Figure 19b, Figure 19f, and Figure 19j, respectively. Figure 19c, Figure 19g, and Figure 19k describe the initial state of the unloading process of the second, third, and fourth cycles, respectively. The initial state of the unloading–relaxation processes of the second, third, and fourth cycles are presented in Figure 19d, Figure 19h, and Figure 19l, respectively. Figure 19m is the ending state of the fourth cycle. Figure 19e and Figure 19i also describe the ending state of the second and third cycles, respectively.

From the analysis of Figure 19, four conclusions were drawn. Firstly, as shown in Figure 19, it can be seen that the transition bonds with a (2 1 0) crystal orientation in area I, area II, and area III existed and remained stable during the three cycles. The crystal structure of the second, third, and fourth cycles showed a good stability performance. In addition, forward and reverse phase transformation occurred in the three transition bonds with a (2 1 0) crystal orientation. Figure 19c,e,g,i,k,l describe the crystal structure after the relaxation processes. Compared with the previous processes, the structure of the three transition bonds was optimized and recovered due to the dynamic recovery effect. Thus, the cyclic training benefited the stability of the three transition bonds.

### 3.3. Cyclic Stability of eCEs Performance

From the above analysis, the Fe14.6Ni(at%) alloy showed great potential for industrial application. In addition, the thermal fatigue life was an essential parameter of the eCEs performance. Therefore, in this section, the thermal fatigue MD simulation adopted the pulse cyclic loading method to investigate cyclic stability. The thermal fatigue MD simulation was performed in the same subinterval and stress pulse as in Section 3.2. Before the thermal fatigue MD simulation started, the preload and the four-time pulse cyclic processes were conducted to train the Fe14.6Ni(at%) alloy to stabilize the eCEs performance.

**Figure 20:**
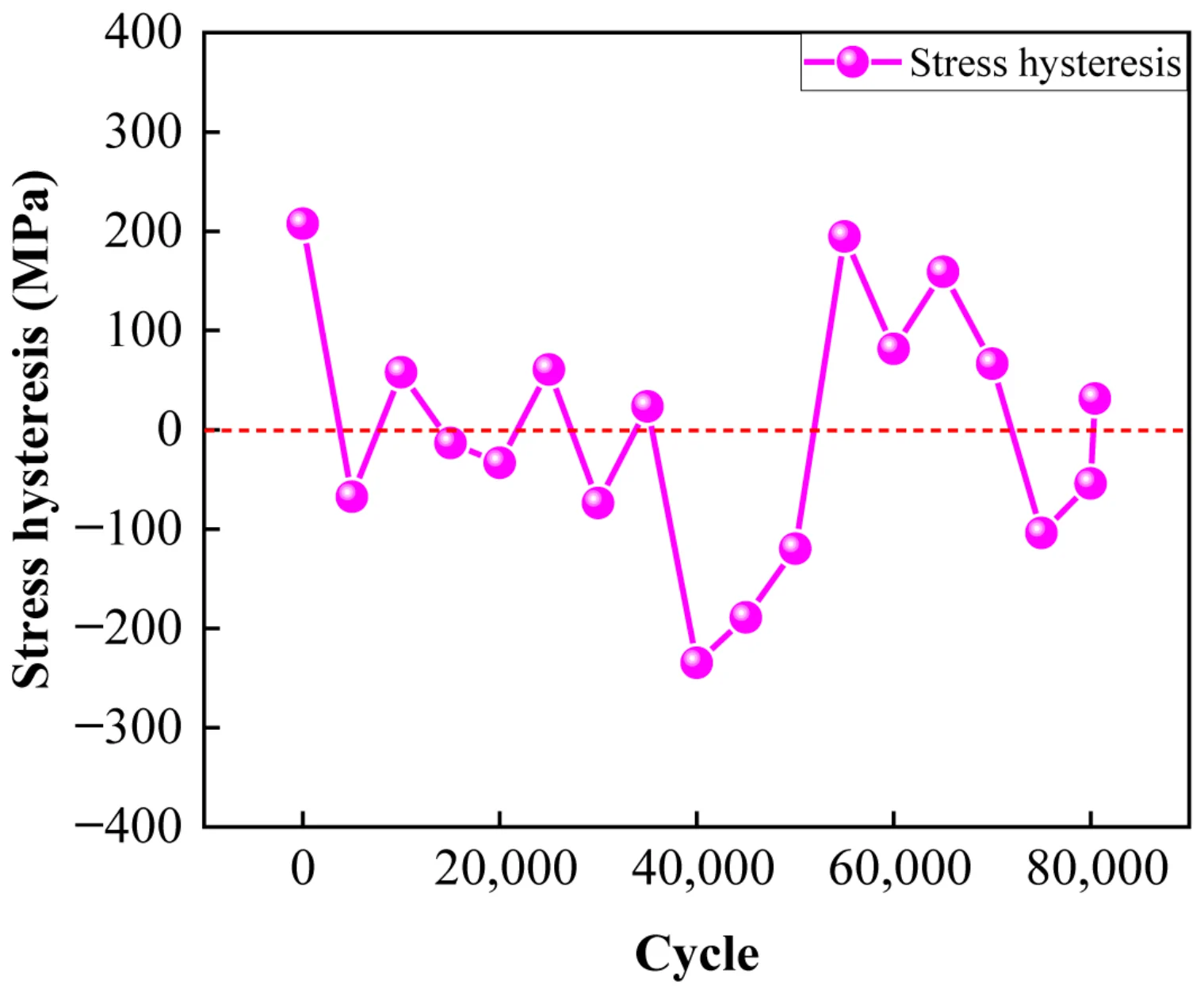
The trend of stress hysteresis with increasing cycles.

**Figure 21:**
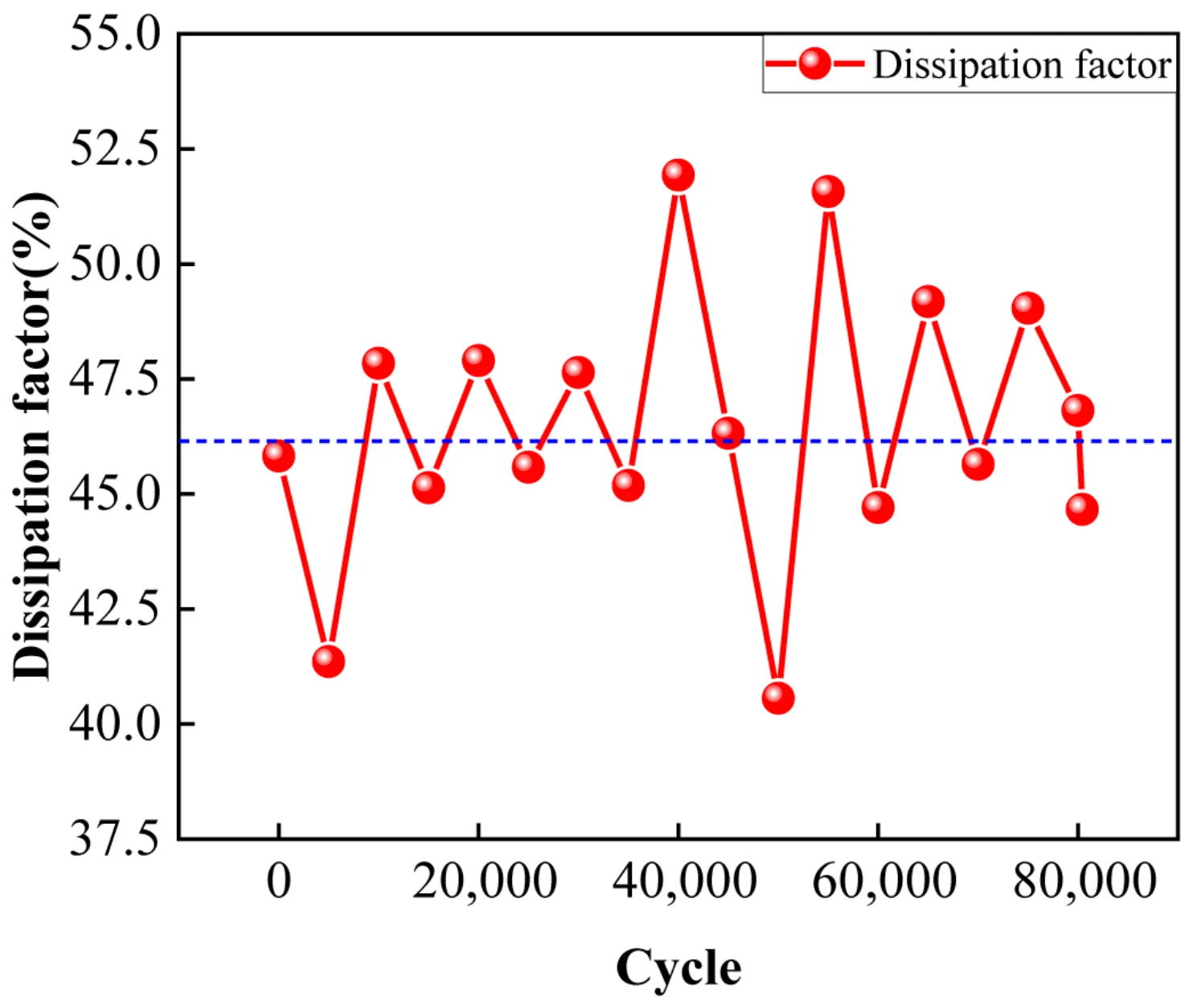
The trend of the Df with increasing cycles.

The stress–strain curves during the thermal fatigue cycles are shown in Figure S6, indicating that the loading and unloading stress–strain curves are the classic parallelogram shape even after the 80414th cycle. This phenomenon illustrated that the stress and strain showed a classic and stable relationship during the thermal fatigue cycles. The stress hysteresis during thermal fatigue cycles is evaluated. Figure 20 shows the trend of the stress hysteresis with increasing cycles. It can be seen that the stress hysteresis fluctuated around a balanced value, within the range of [−234.82 MPa, 207.63 MPa], remaining at around a ±1.5% fluctuation in the thermal fatigue cycles, which presented an outstanding stability and a small stress hysteresis. Figure 21 presents the trend of the *D_f_* with increasing cycles. From Figure 21, it can be seen that the *D_f_* showed a fluctuating trend, remaining around 45% with increasing cycles, which presents a stable performance of the energy dissipation.

**Figure 22:**
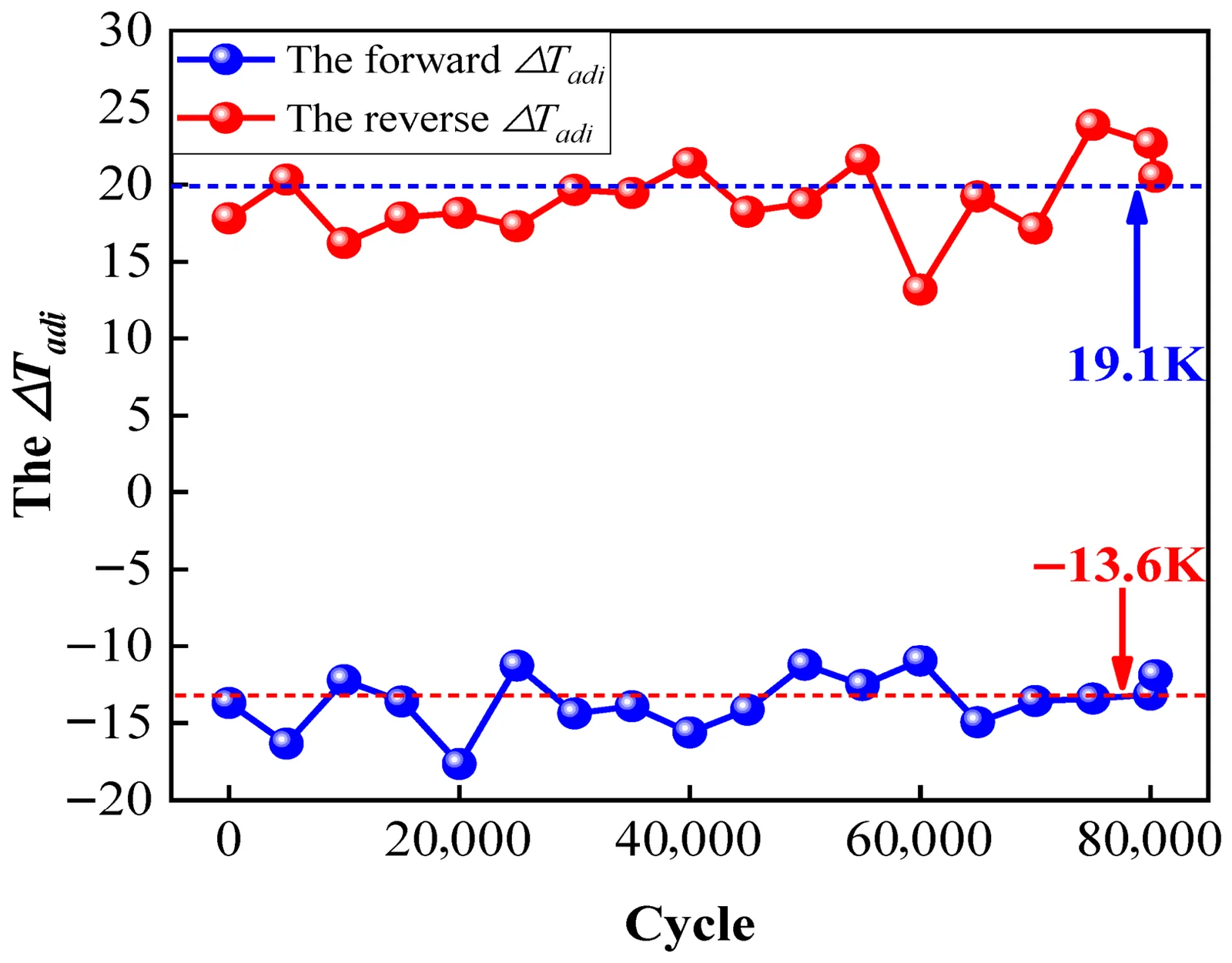
The trend of the ∆T_adi_ with increasing cycles.

Figure S7 depicts the temperature trend during the thermal fatigue cycles. As shown in Figure S7, the temperature shows a typical variation even after 80,414 cycles, proving the stability of the trend. This was vital not only for stable eCEs performance but also for potential practical applications. The ∆T_adi_ during the loading and unloading processes is plotted in Figure 22. It can be seen that the forward ∆T_adi_ fluctuated around −13.6 K, and the reverse ∆T_adi_ fluctuated around 19.1 K, which showed the excellent performance of the ∆T_adi_ and the reliability of the of Fe14.6Ni(at%) alloy.

**Figure 23:**
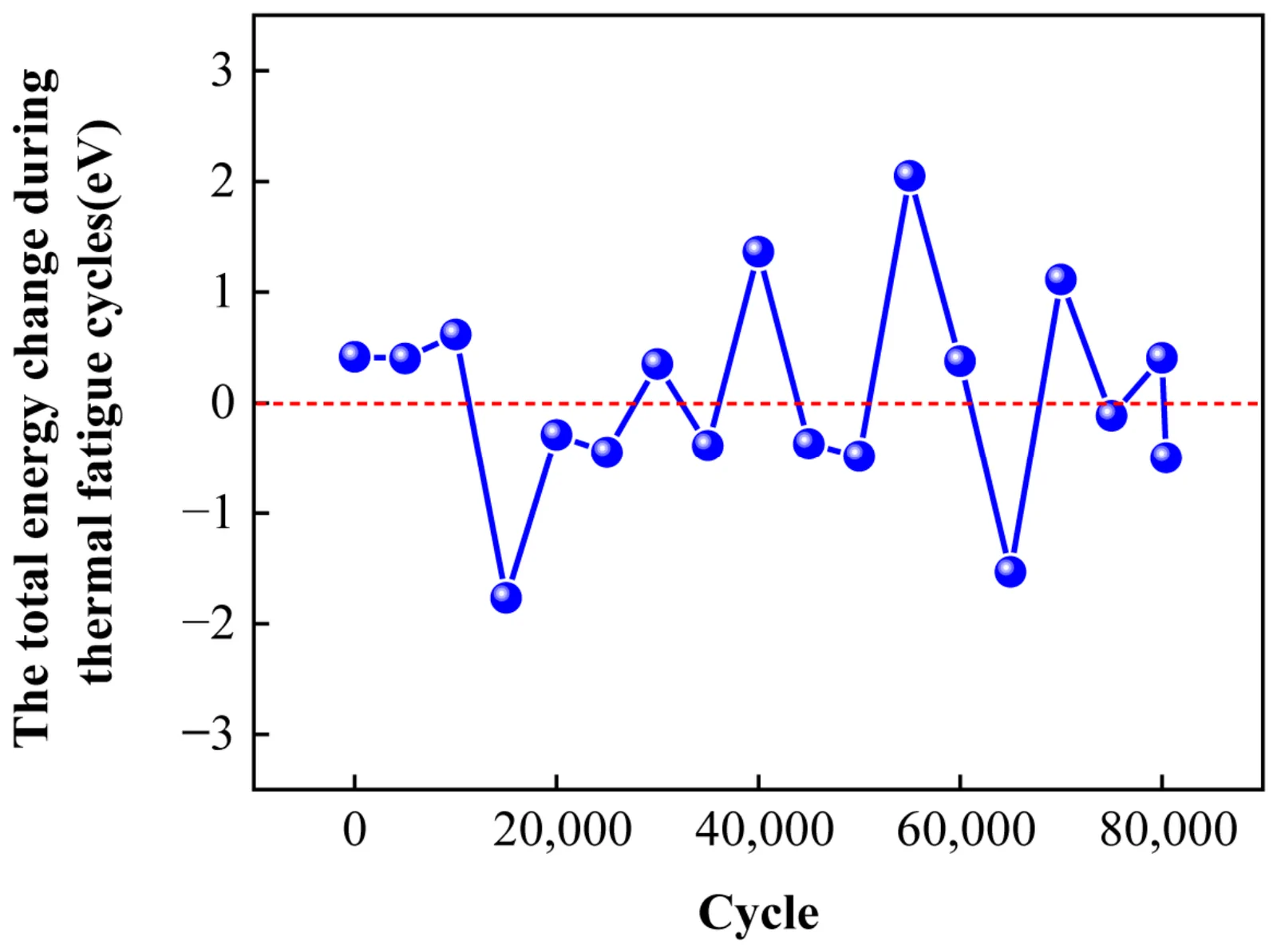
The trend of the total energy change with increasing cycles.

Figure S8 shows the total energy trend during thermal fatigue cycles. From Figure S8, it can be seen that the total energy showed a typical shape even after 80,414 cycles, which presents the steady performance of the total energy during the thermal fatigue cycles. Moreover, the total energy change during the thermal fatigue cycles is pictured in Figure 23, demonstrating that total energy change fluctuated around zero. Within the range of [−1.77 eV, 2.05 eV], the brilliant performance of the stabilization of the total energy can be observed.

**Figure 24:**
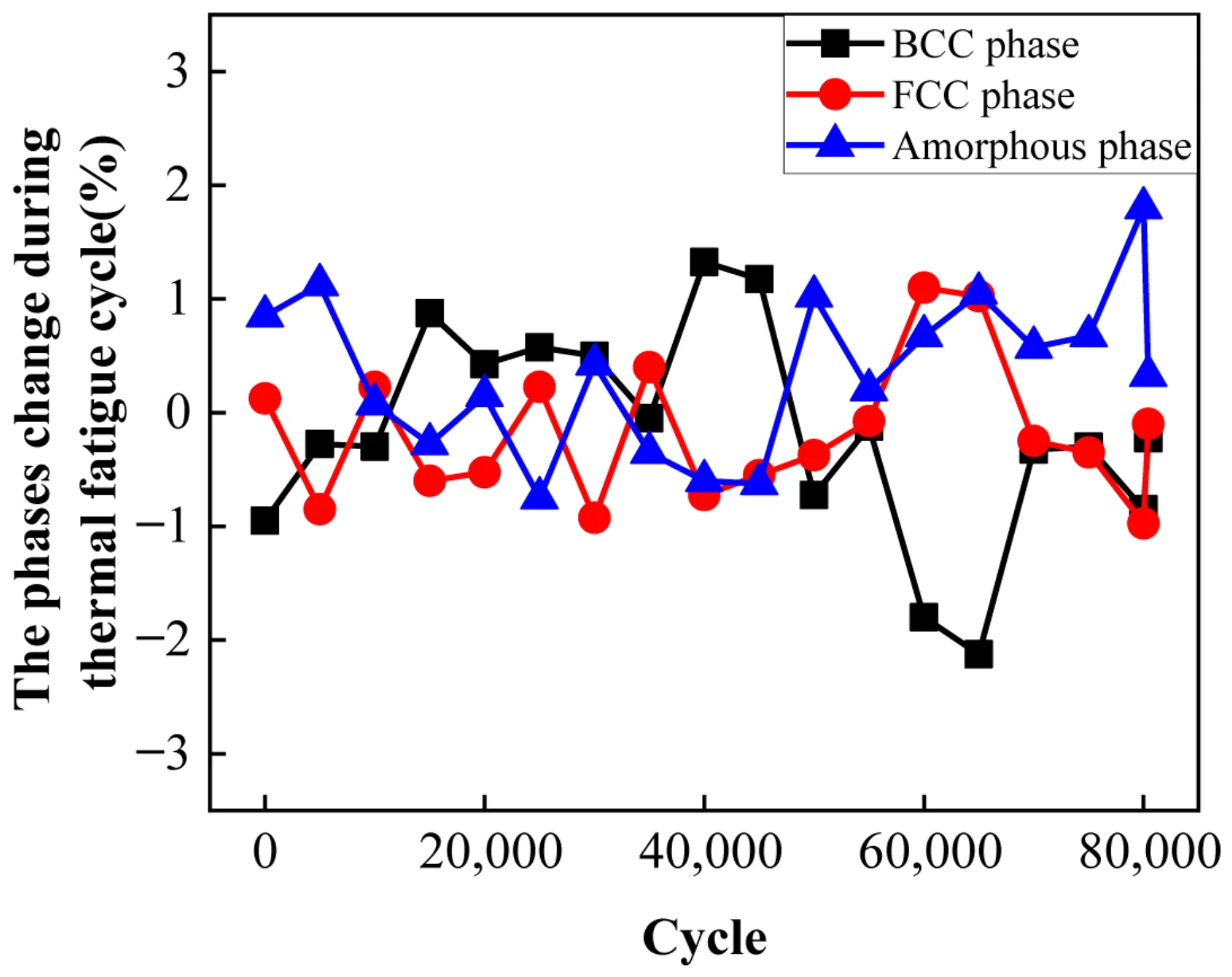
The trend of the three phase content changes with increasing cycles.

Figures S9–S11 present the variation of the content of BCC phase, FCC phase, and transition amorphous phase during thermal fatigue cycles, respectively. As shown in Figures S9–S11, the curves of the BCC phase, FCC phase, and transition amorphous phase present a typical variation, as illustrated in Section 3.2. This phenomenon demonstrates that the phase transformation process steadily progressed even when the cycles increased to 80,414. This is why the root causes of stress, stress hysteresis, *D_f_*, temperature, ∆T_adi_, and total energy varied at a constant level during the thermal fatigue cycles. The phase content change can be used to describe the stability of the cycle. Figure 24 shows the content changes in the BCC phase, the FCC phase, and the transition amorphous phase during thermal fatigue cycles. From Figure 24, it can be seen that the BCC phase, FCC phase, and transition amorphous phase varied at an extremely small level during a cycle and remained within the range of ±2%, ±1%, and ±2%, respectively. This phenomenon indicates that the three phases’ content remained at a stable level even after 80,414 cycles. Furthermore, the crystal structure states of the Fe14.6Ni(at%) alloy at the end of the thermal fatigue cycles are shown in Figure S12. Figure S12 indicates that the three transition bonds are stably maintained at the (2 1 0) crystal orientation and the five-layer structure of “‘BCC’-‘transition amorphous’-‘FCC’-‘transition amorphous’-‘BCC’” is stable, which demonstrates that the transition bonds remained sensitive to the external stimuli even after 80,414 cycles.

In summary, the results show that the stress, temperature, stress hysteresis, dissipation factor, temperature, content of phases, and crystal structure distribution behaved regularly. The eCEs performance of the Fe14.6Ni(at%) alloy remains sensitive to the external stimuli even after 80,414 cycles. The thermal fatigue life is two or three orders of magnitude greater than the Fe-based alloy that has been reported [13], which depicts a huge potential for industrial applications.

In subsequent work, high-temperature solution treatment and low-temperature heat treatment processes will be adapted to prepare the Fe14.6Ni single crystal alloy. In comparison with the MD simulation results, then, uniaxial tensile experiments will be conducted to test the fatigue life during successive loading and pulsing cyclic loading processes. At the same time, according to the adiabatic temperature change results gained from the MD simulation, infrared thermal imaging will be used to monitor the adiabatic temperature change and the temperature field evolution process during successive loading and pulsing cyclic loading processes. The subsequent work will further confirm the MD simulation results and show the promising industrial application prospects.

## 4. Conclusions

In this investigation, successive and pulsing loading methods were employed to evaluate the eCEs performance of the Fe14.6Ni(at%) alloy at 293 K by MD simulation. The study of the loading methods indicated that the four-time successive cyclic loading method was not the favorable option due to the significant stress hysteresis, the chaos and unstable state of temperature, the consumption of the BCC phase, and the disordered crystal structure distribution, even though this method produced a small *D_f_*. Compared with the successive cyclic loading method, the results showed that the pulse cyclic loading method was the optimal option due to the regular evolution processes of the stress, temperature, content of the phases, and crystal structure, even though this method depicted a larger *D_f_* than the successive cyclic loading method. Finally, the research on cyclic stability showed that the Fe14.6Ni(at%) alloy remained sensitive to the external stimuli even after 80,414 cycles. The thermal fatigue life was two or three orders of magnitude greater than the Fe-based alloy that has been reported. The work demonstrates the great industrial potential of the Fe14.6Ni(at%) alloy and proposes a promising strategy for optimizing the eCEs performance.

## Data Availability

The original contributions presented in this study are included in the article/Supplementary Material. Further inquiries can be directed to the corresponding author.

## Supplementary Materials

The following supporting information can be downloaded at: https://www.mdpi.com/article/10.3390/ma19183909/s1, Figure S1. The stress-strain curve of Fe14.6Ni(at%) alloy at 293 K under strain rate of 0.3 ps^−1^. Table S1. The six-stage strain intervals of stress at 293 K under strain rate of 0.3 ps^−1^. Figure S2. The stress-strain curves during the third and the fourth successive cyclic loading processes. (a) The third cycle; (b) The fourth cycle. Figure S3. The energy dissipation of the Fe14.6Ni(at%) alloy during the third and the fourth successive cyclic loading processes. (a) The third cycle; (b) The fourth cycle. Figure S4. The stress-strain curves during the third and the fourth pulse cyclic loading processes. (a) The third cycle; (b) The fourth cycle. Figure S5. The energy dissipation of the Fe14.6Ni(at%) alloy during the third and the fourth pulse cyclic loading processes. (a) The third cycle; (b) The fourth cycle. Figure S6. The stress-strain curves during the thermal fatigue cycles. (a) 1st~15000th cycles; (b) 20000th~35000th cycles; (c) 40000th~60000th cycles; (d) 65000th~80414th cycles. Figure S7. The temperature variation trend during the thermal fatigue cycles. (a) 1st~15000th cycles; (b) 20000th~35000th cycles; (c) 40000th~60000th cycles; (d) 65000th~80414th cycles. Figure S8. The total energy variation trend during thermal fatigue cycles. (a) 1st~15000th cycles; (b) 20000th~35000th cycles; (c) 40000th~60000th cycles; (d) 65000th~80414th cycles. Figure S9. The variation trend of the content of BCC phase during the thermal fatigue cycles. (a) 1st~15000th cycles; (b) 20000th~35000th cycles; (c) 40000th~60000th cycles; (d) 65000th~80414th cycles. Figure S10. The variation trend of the content of FCC phase during the thermal fatigue cycles. (a) 1st~15000th cycles; (b) 20000th~35000th cycles; (c) 40000th~60000th cycles; (d) 65000th~80414th cycles. Figure S11. The variation trend of the content of transition amorphous phase during the thermal fatigue cycles. (a) 1st~15000th cycles; (b) 20000th~35000th cycles; (c) 40000th~60000th cycles; (d) 65000th~80414th cycles. Figure S12. The crystal structure states of the Fe14.6Ni(at%) alloy at the end during the thermal fatigue cycles. (a) The 1st; (b) The 5000th; (c) The 10000th; (d) The 15000th; (e) The 20000th; (f) The 25000th; (g) The 30000th; (h) The 35000th; (i) The 40000th; (j) The 45000th; (k) The 50000th; (l) The 55000th; (m) The 60000th; (n) The 65000th; (o) The 70000th; (p) The 75000th; (q) The 80000th; (r) The 80414th.
