# Supplementary material for: Research on the Elastocaloric Effects Performance of the Fe14.6Ni(at%) Alloy via Successive and Pulsing Loading Methods: A Molecular Dynamics Study
Source: Materials (Basel). 2026 Sep 15;19(18):3909. doi: 10.3390/ma19183909 (PMC13608735; doi:10.3390/ma19183909)
Supplement: Supplementary file 1 [file materials-19-03909-s001.zip › materials-3923482-supplementary.pdf]

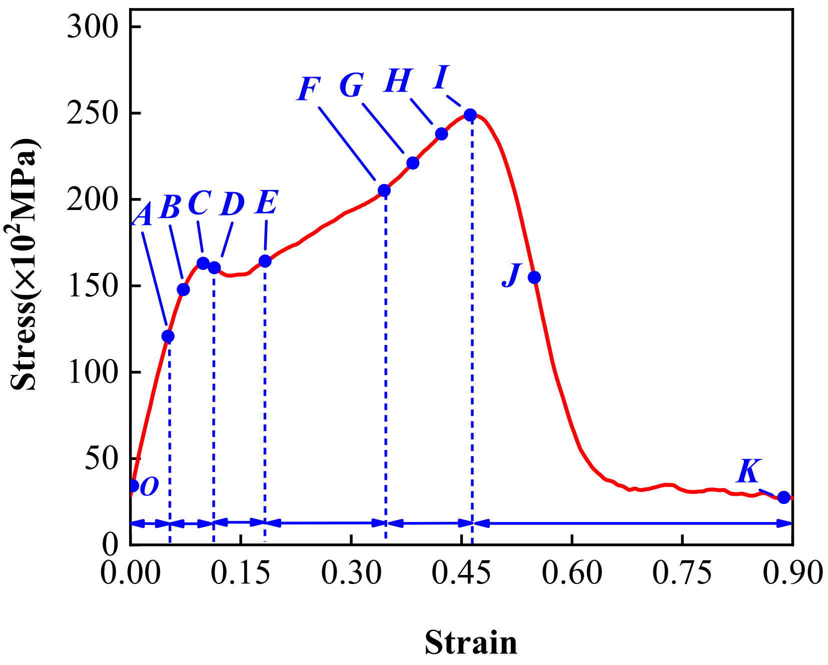

Figure S1. The stress-strain curve of Fe14.6Ni(at%) alloy at 293K under strain rate of 0.3ps^{-1}.

Table S1. The six-stage strain intervals of stress at 293K under strain rate of 0.3ps^{-1}.

**Stages**	**Strain intervals**
Superelastic stage	[0.000,0.063]
Superelastic yield stage	[0.063,0.108]
Phase transformation stage	[0.108,0.177]
Elastic stage	[0.177,0.381]
Yield stage	[0.381,0.468]
Fracture stage	[0.468,0.900]

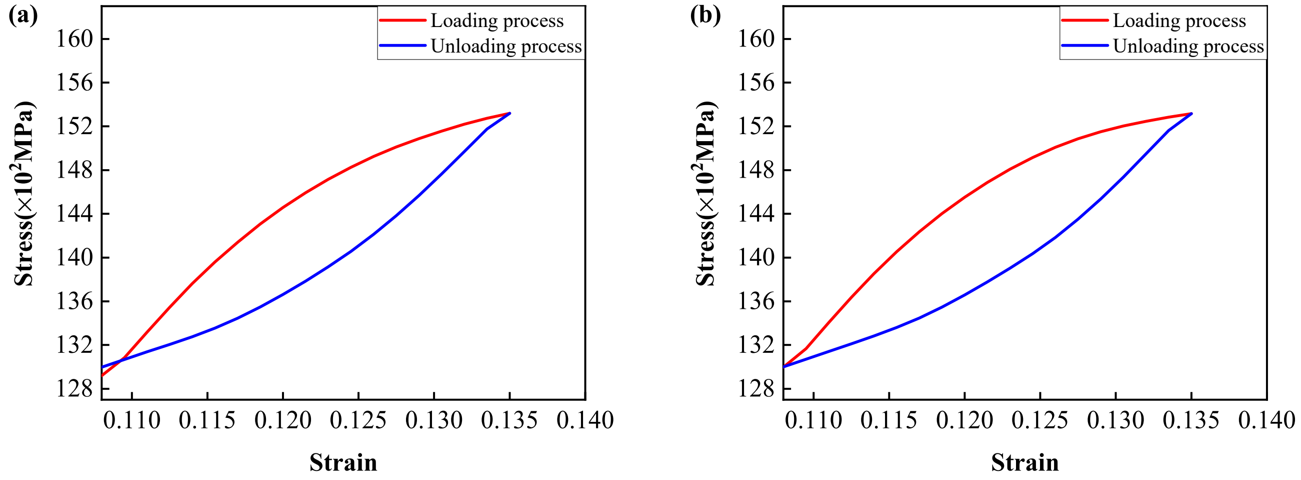

Figure S2. The stress-strain curves during the third and the fourth successive cyclic loading processes. (a) The third cycle; (b) The fourth cycle.

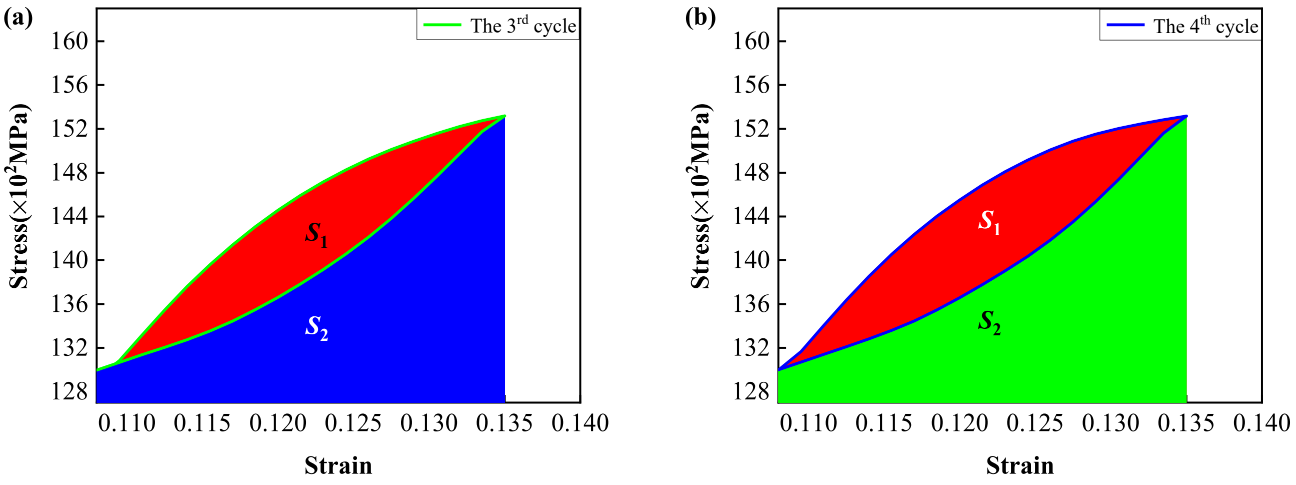

Figure S3. The energy dissipation of the Fe14.6Ni(at%) alloy during the third and the fourth successive cyclic loading processes. (a) The third cycle; (b) The fourth cycle.

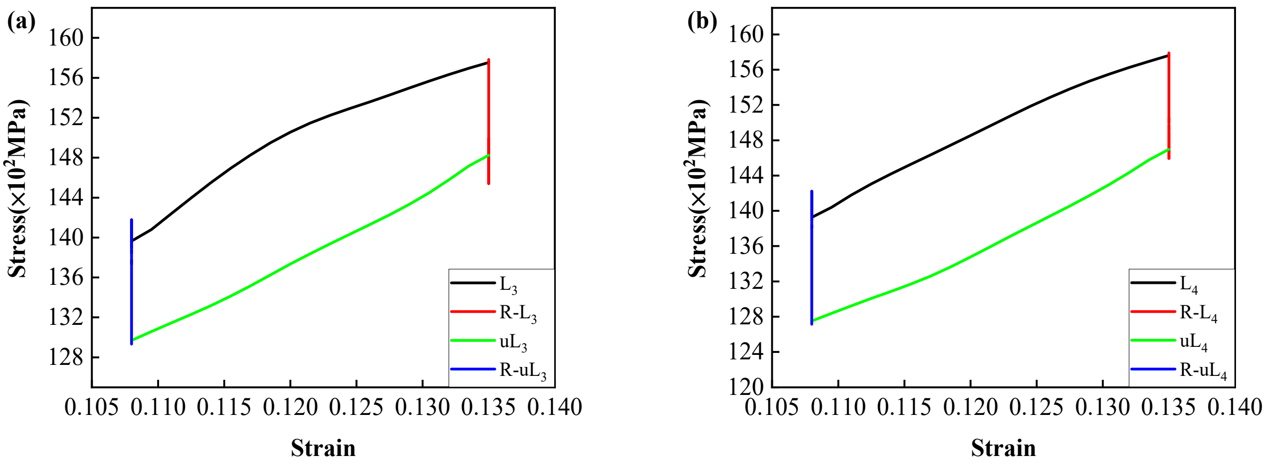

Figure S4. The stress-strain curves during the third and the fourth pulse cyclic loading processes. (a) The third cycle; (b) The fourth cycle.

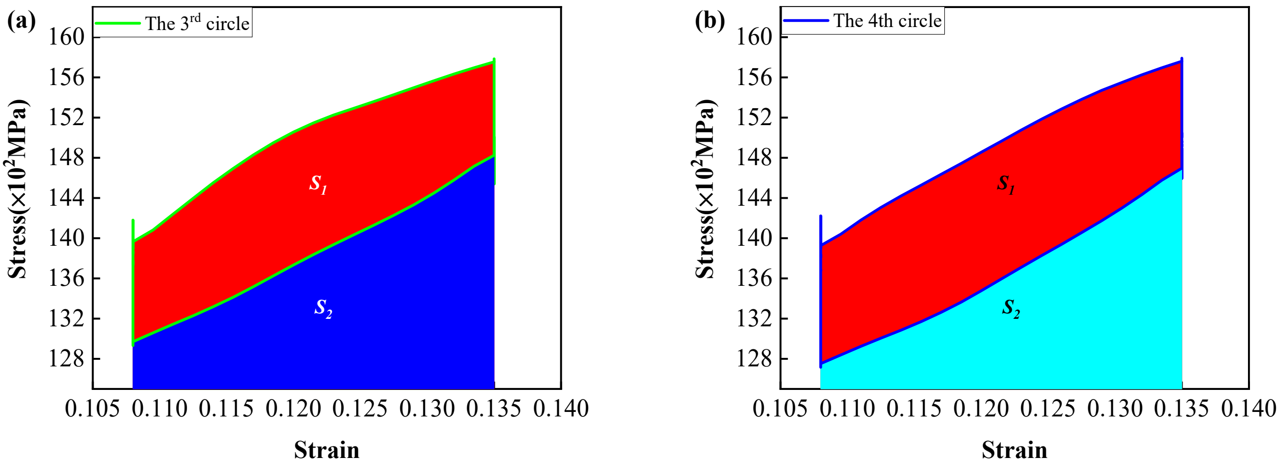

Figure S5. The energy dissipation of the Fe14.6Ni(at%) alloy during the third and the fourth pulse cyclic loading processes. (a) The third cycle; (b) The fourth cycle.

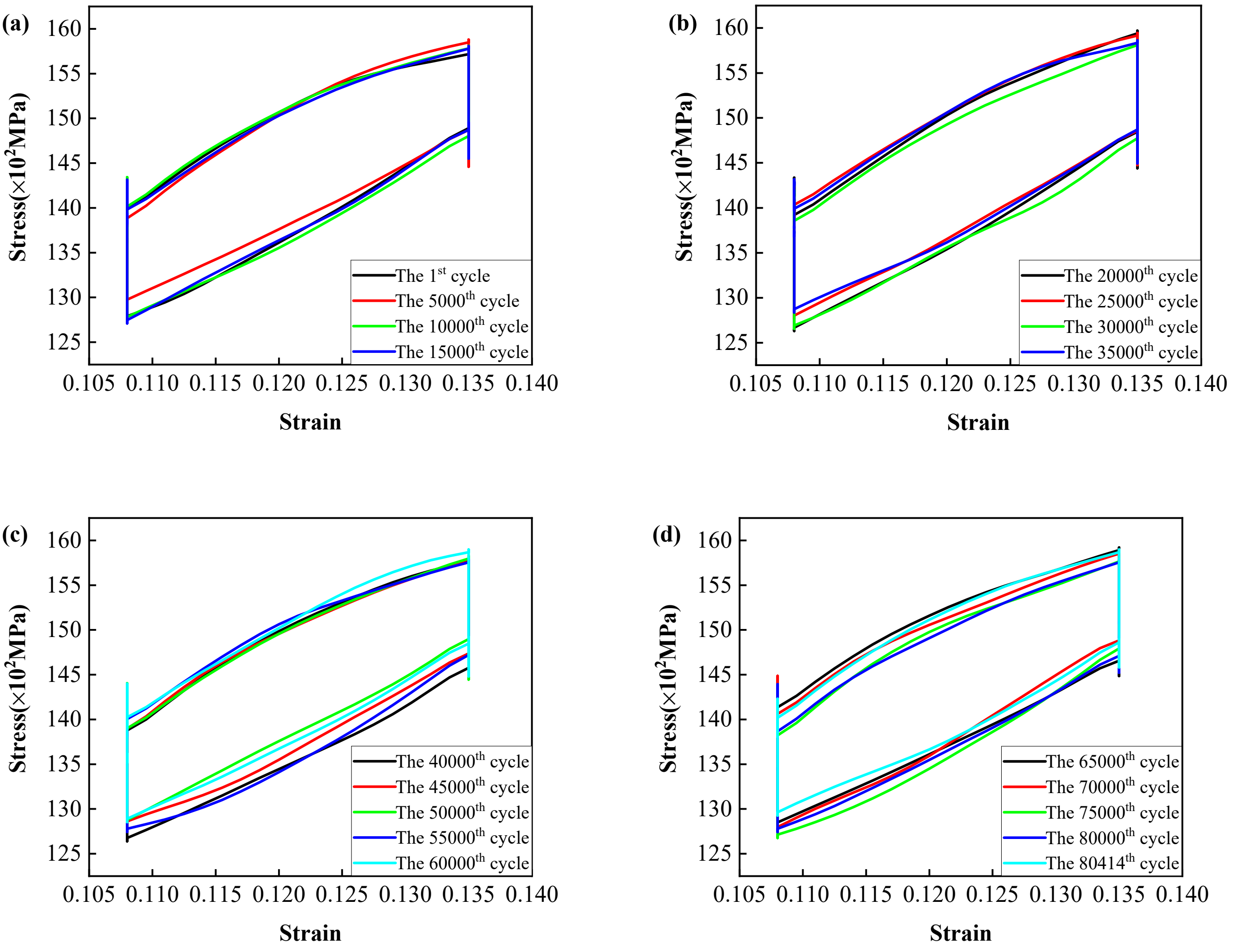

Figure S6. The stress-strain curves during the thermal fatigue cycles. (a) 1st ~ 15000th cycles; (b) 20000th ~ 35000th cycles; (c) 40000th ~ 60000th cycles; (d) 65000th ~ 80414th cycles.

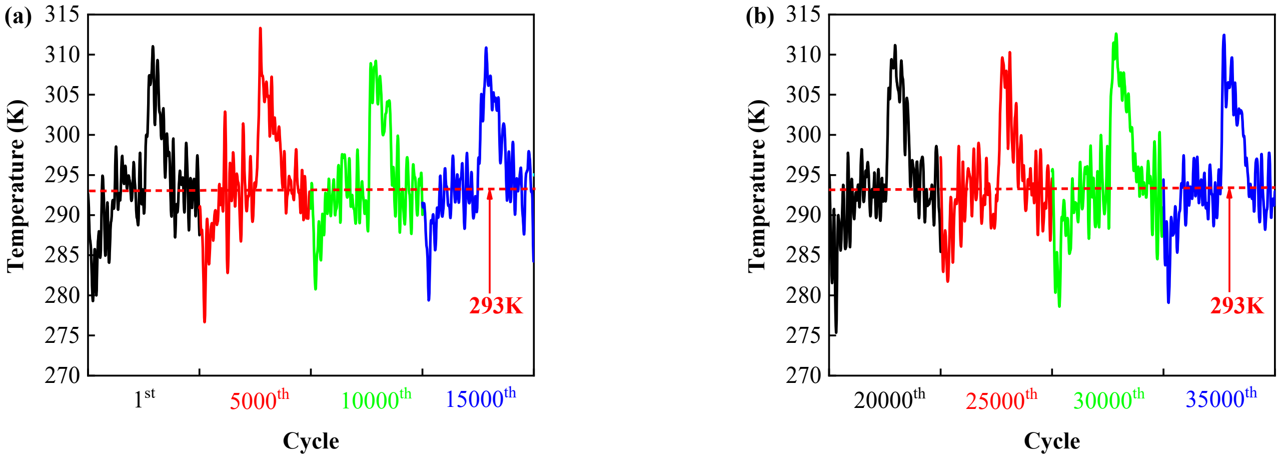

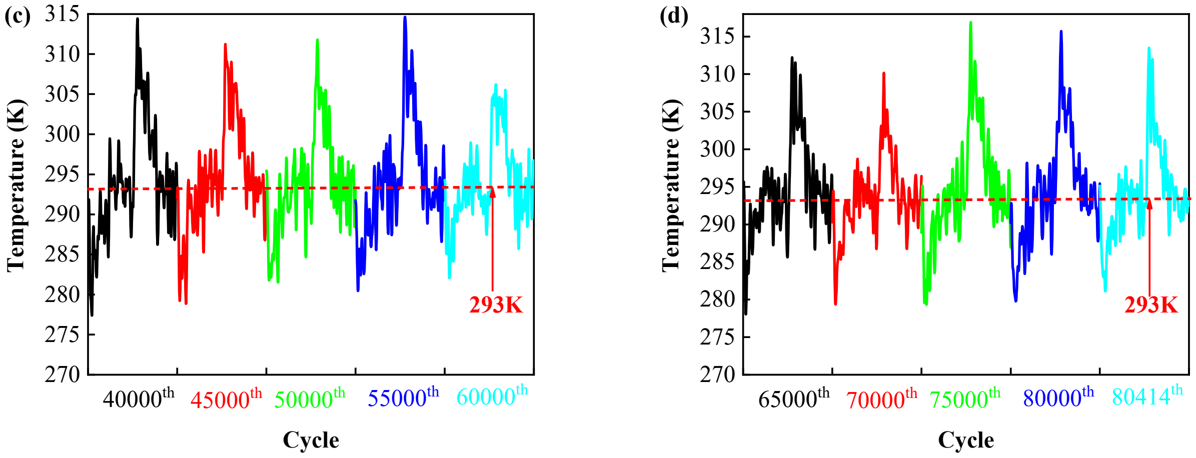

Figure S7. The temperature variation trend during the thermal fatigue cycles. (a) 1st ~ 15000th cycles; (b) 20000th ~ 35000th cycles; (c) 40000th ~ 60000th cycles; (d) 65000th ~ 80414th cycles.

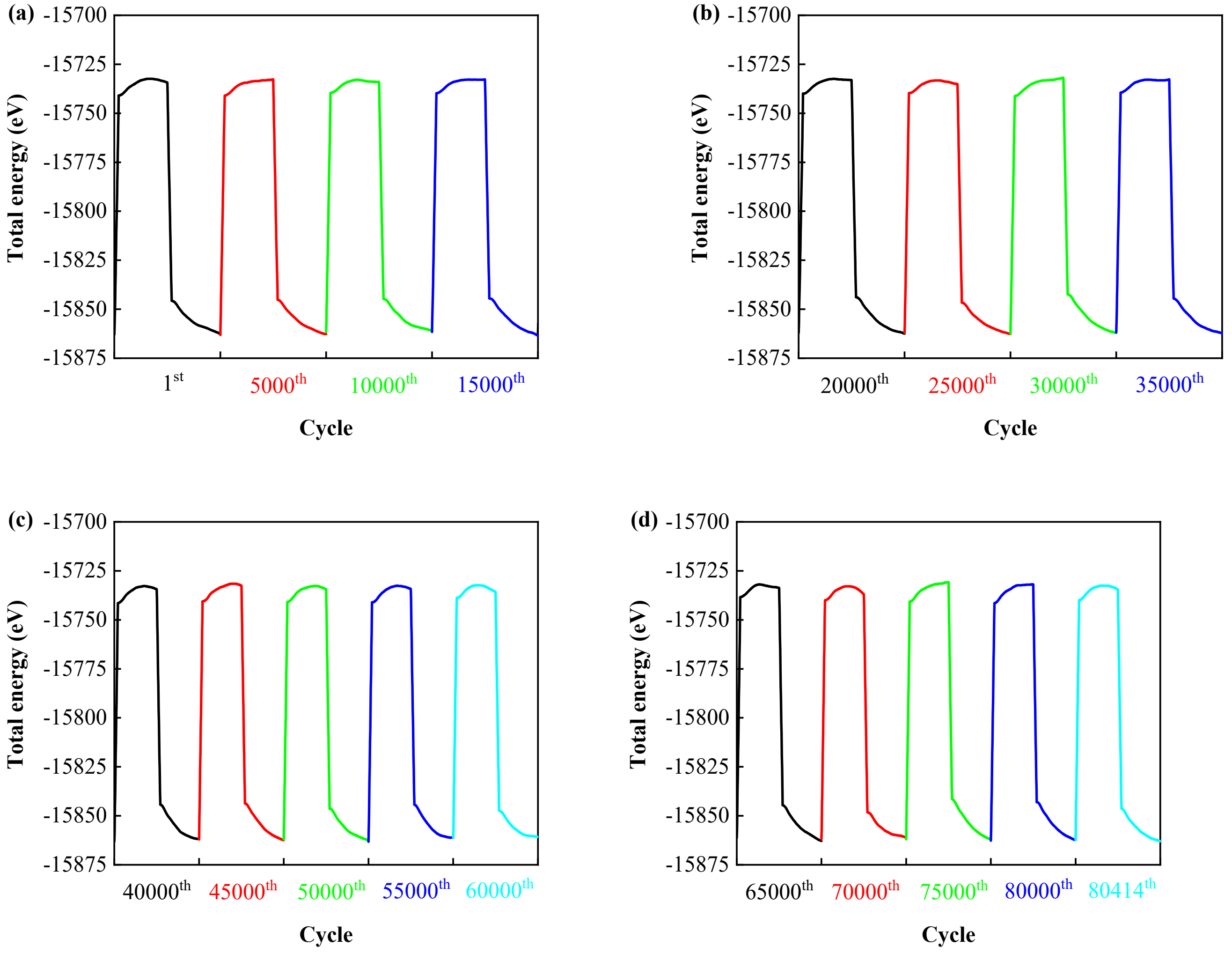

Figure S8. The total energy variation trend during thermal fatigue cycles. (a) 1st ~ 15000th cycles; (b) 20000th ~ 35000th cycles; (c) 40000th ~ 60000th cycles; (d) 65000th ~ 80414th cycles.

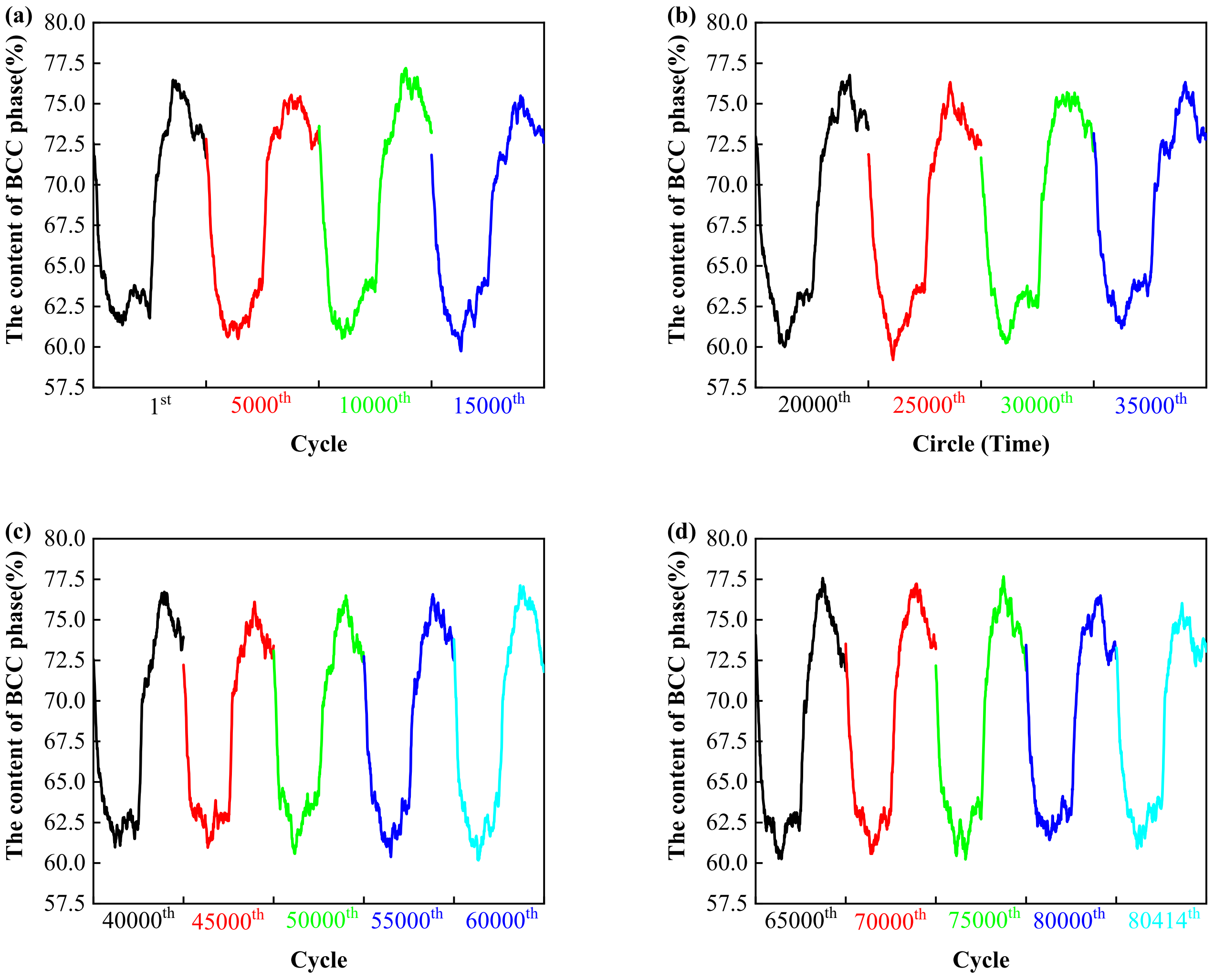

Figure S9. The variation trend of the content of BCC phase during the thermal fatigue cycles. (a) 1st ~ 15000th cycles; (b) 20000th ~ 35000th cycles; (c) 40000th ~ 60000th cycles; (d) 65000th ~ 80414th cycles.

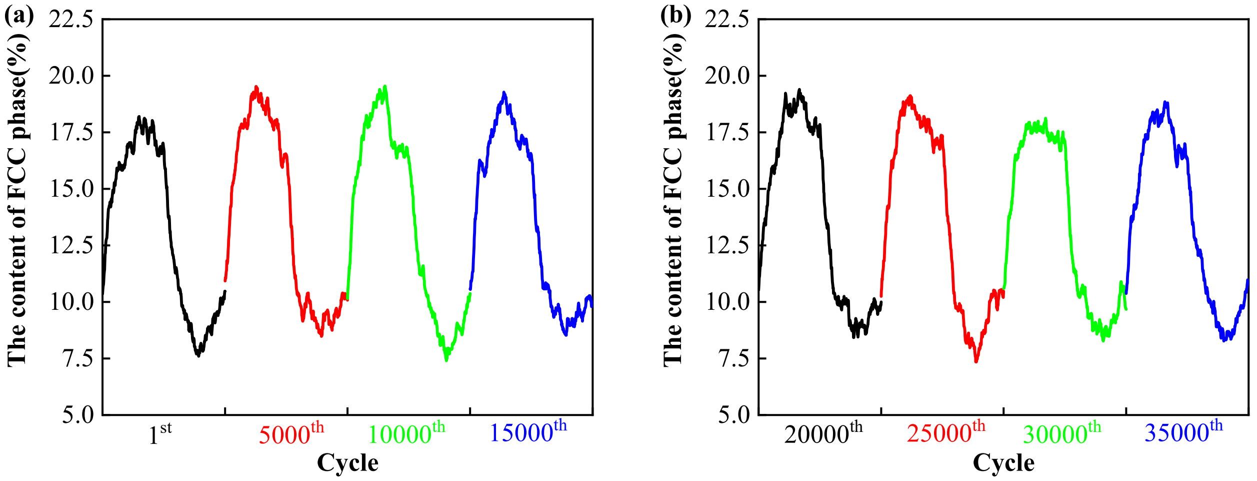

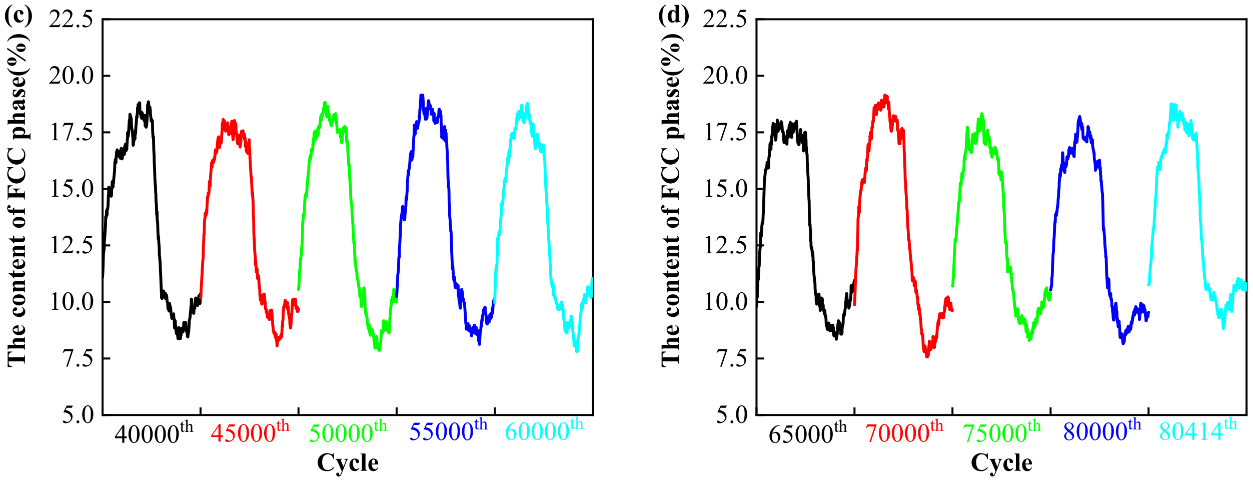

Figure S10. The variation trend of the content of FCC phase during the thermal fatigue cycles. (a) 1st ~ 15000th cycles; (b) 20000th ~ 35000th cycles; (c) 40000th ~ 60000th cycles; (d) 65000th ~ 80414th cycles.

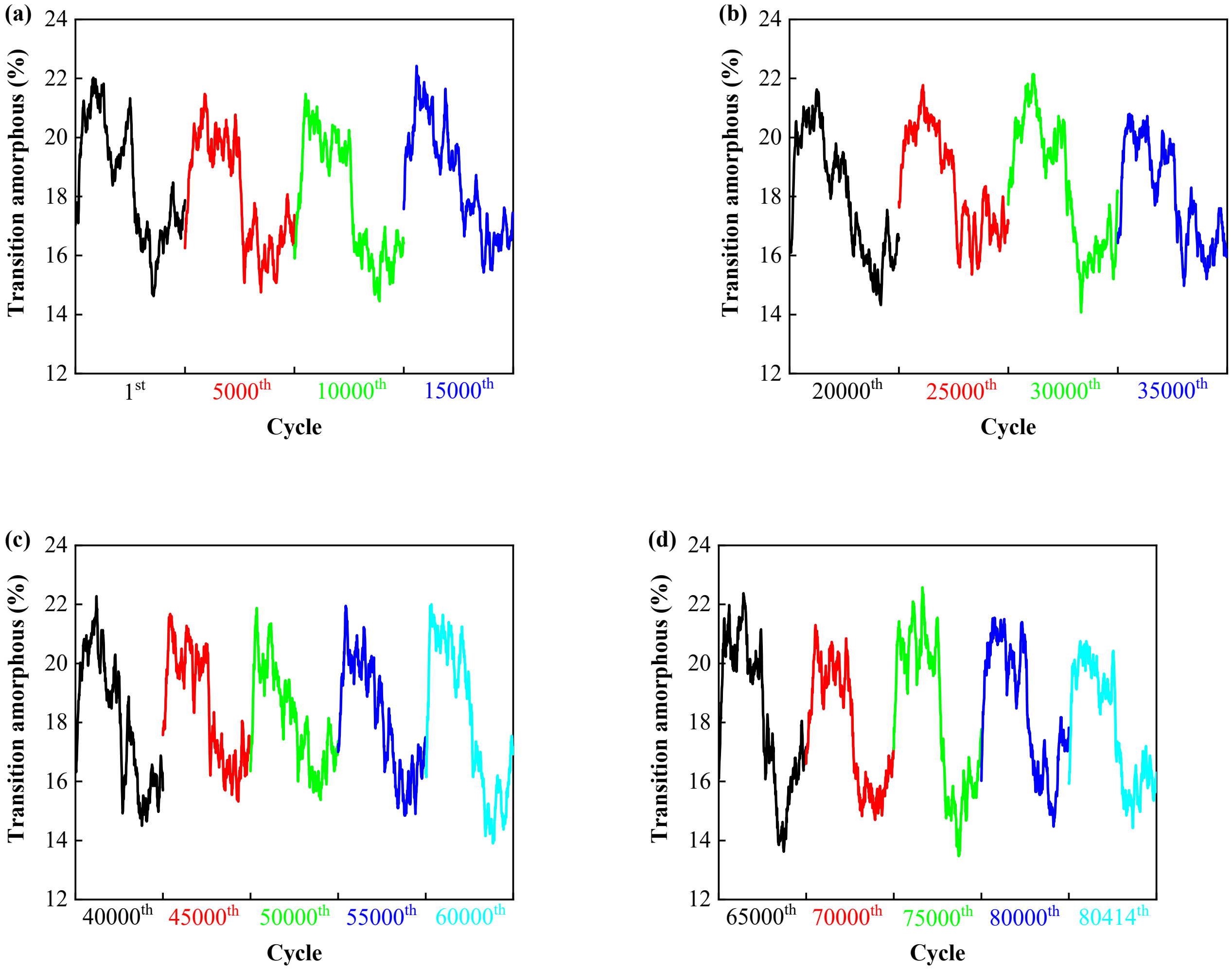

Figure S11. The variation trend of the content of transition amorphous phase during the thermal fatigue cycles. (a) 1st ~ 15000th cycles; (b) 20000th ~ 35000th cycles; (c) 40000th ~ 60000th cycles; (d) 65000th ~ 80414th cycles.

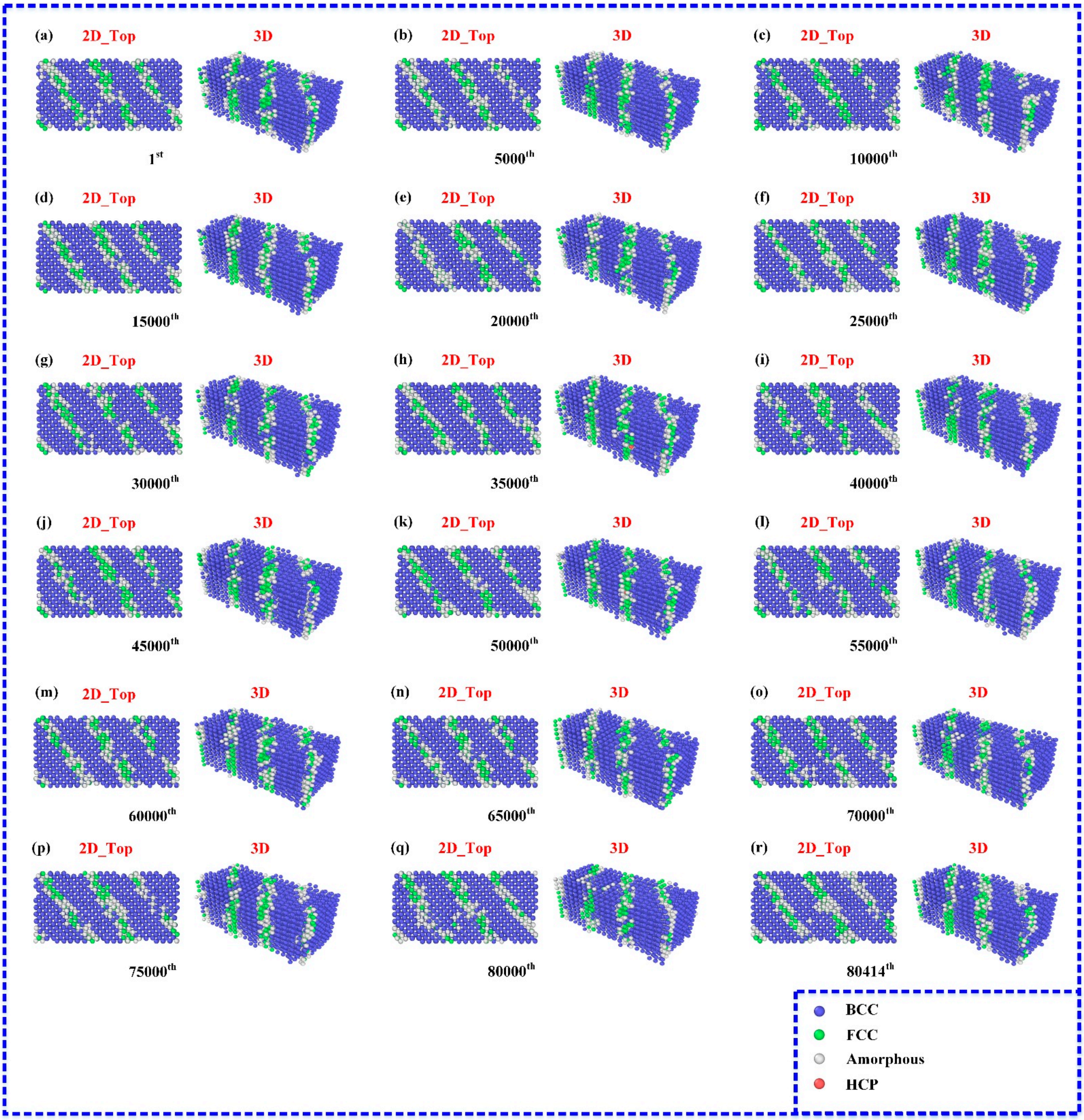

Figure S12. The crystal structure states of the Fe14.6Ni(at%) alloy at the end during the thermal fatigue cycles. (a)The 1st; (b)The 5000th; (c) The 10000th; (d) The 15000th; (e) The 20000th; (f) The 25000th; (g) The 30000th; (h) The 35000th; (i) The 40000th; (j) The 45000th; (k) The 50000th; (l) The 55000th; (m) The 60000th; (n) The 65000th; (o) The 70000th; (p) The 75000th; (q) The 80000th; (r) The 80414th.
